# Genome-wide association studies of antidepressant class response and treatment-resistant depression

**DOI:** 10.1038/s41398-020-01035-6

**Published:** 2020-10-26

**Authors:** Qingqin S. Li, Chao Tian, David Hinds, 23andMe Research Team, Michelle Agee, Babak Alipanahi, Adam Auton, Robert K. Bell, Katarzyna Bryc, Sarah L. Elson, Pierre Fontanillas, Nicholas A. Furlotte, Karen E. Huber, Aaron Kleinman, Nadia K. Litterman, Matthew H. McIntyre, Joanna L. Mountain, Elizabeth S. Noblin, Carrie A. M. Northover, Steven J. Pitts, J. Fah Sathirapongsasuti, Olga V. Sazonova, Janie F. Shelton, Suyash Shringarpure, Joyce Y. Tung, Vladimir Vacic, Catherine H. Wilson, Amir S. Zare

**Affiliations:** 1 Janssen Research & Development, LLC, Titusville, NJ USA; 2 23andMe, Inc., Sunnyvale, CA USA

**Keywords:** Genomics, Clinical genetics

## Abstract

The “antidepressant efficacy” survey (AES) was deployed to > 50,000 23andMe, Inc. research participants to investigate the genetic basis of treatment-resistant depression (TRD) and non-treatment-resistant depression (NTRD). Genome-wide association studies (GWAS) were performed, including TRD vs. NTRD, selective serotonin reuptake inhibitor (SSRI) responders vs. non-responders, serotonin-norepinephrine reuptake inhibitor (SNRI) responders vs. non-responders, and norepinephrine-dopamine reuptake inhibitor responders vs. non-responders. Only the SSRI association reached the genome-wide significance threshold (*p* < 5 × 10^−8^): one genomic region in *RNF219-AS1* (SNP rs4884091, *p* = 2.42 × 10^−8^, OR = 1.21); this association was also observed in the meta-analysis (13,130 responders vs. 6,610 non-responders) of AES and an earlier “antidepressant efficacy and side effects” survey (AESES) cohort. Meta-analysis for SNRI response phenotype derived from AES and AESES (4030 responders vs. 3049 non-responders) identified another genomic region (lead SNP rs4955665, *p* = 1.62 × 10^−9^, OR = 1.25) in an intronic region of *MECOM* passing the genome-wide significance threshold. Meta-analysis for the TRD phenotype (31,068 NTRD vs 5,714 TRD) identified one additional genomic region (lead SNP rs150245813, *p* = 8.07 × 10^−9^, OR = 0.80) in 10p11.1 passing the genome-wide significance threshold. A stronger association for rs150245813 was observed in current study (*p* = 7.35 × 10^−7^, OR = 0.79) than the previous study (*p* = 1.40 × 10^−3^, OR = 0.81), and for rs4955665, a stronger association in previous study (*p* = 1.21 × 10^−6^, OR = 1.27) than the current study (*p* = 2.64 × 10^−4^, OR = 1.21). In total, three novel loci associated with SSRI or SNRI (responders vs. non-responders), and NTRD vs TRD were identified; gene level association and gene set enrichment analyses implicate enrichment of genes involved in immune process.

## Introduction

A wide variety of antidepressants are available for major depressive disorder (MDD) and response to treatment varies in time to onset of benefit, overall efficacy, and duration of effect. Approximately 30% of individuals with MDD who are considered to have treatment-resistant depression (TRD) do not achieve full remission despite treatment with multiple agents at an adequate dose and duration^1^. Genetic variability may contribute to the differences in drug-specific, class-specific response, or TRD. Genome wide association studies (GWAS) have been employed as an approach to identify novel genetic variants that may contribute to variations in antidepressant response.

Several antidepressant efficacy GWAS have been conducted using samples from the munich antidepressant response signature (MARS) project (a naturalistic prospective study, *n* = 339)^2^, the genome-based therapeutic drugs for depression (GENDEP) project (*n* = 394 on escitalopram and *n* = 312 on nortriptyline)^3^, the sequenced treatment alternatives to relieve depression (STAR*D) study (*n* = 1491 on citalopram)^4^, the mayo clinic pharmacogenomic research network antidepressant medication pharmacogenomic study (PGRN-AMPS) study (*n* = 529 individuals on selective serotonin reuptake inhibitors [SSRI])^5^, and the Janssen-23andMe antidepressant efficacy GWAS study^6^.

No significant genome-wide associations were found in the analysis of individual-level data from the novel methods leading to new medications in depression and schizophrenia (NEWMEDS) consortium, which consisted of 1790 individuals of European-ancestry with MDD; nor in the meta-analysis of the NEWMEDS and STAR*D studies (*n* = 2,897)^7^. In the antidepressant efficacy GWAS meta-analysis performed on three studies with data from individuals of Northern European descent (STAR*D, GENDEP, and MARS [*n* = 2256]), no variants passing the genome-wide significance threshold associated with antidepressant response were identified in primary outcome assessment of percentage improvement on clinician-rated depression scales and remission rates after 12 weeks of treatment^8^. Recently, Fabbri et al. re-analyzed GENDEP and STAR*D samples by adding the exome array rare variant content and using the Haplotype Reference Consortium (HRC) panel for imputation and identified rs116692768 (*p* = 1.80 × 10^−8^), integrin subunit alpha 9 (*ITGA9*) and rs76191705 (*p* = 2.59 × 10^−8^), neurexin 3 (*NRXN3*) to be significantly associated with symptom improvement during citalopram/escitalopram treatment^9^. Only the association between rs116692768 and symptom improvement was replicated in PGRN-AMPS (*p* = 0.047) and neither polymorphism was replicated in NEWMEDS^9^. Lastly, multi-allelic polygenic risk scores to estimate MDD risk score also showed no prediction of antidepressant treatment response^10^.

The antidepressant response information obtained from self-reported questionnaires could offer an alternative approach to conduct a study with much larger sample sizes. In the current study, treatment outcome data based on an antidepressant efficacy survey (AES)^11^ deployed to 23andMe’s participants were utilized in GWAS. The primary aim of this study was to identify novel genetic variants specifically associated with response to classes of antidepressant therapy to improve our understanding of a potential genetic basis of antidepressant treatment response and to differentiate TRD from non-TRD (NTRD). Furthermore, a similar GWAS using phenotype data derived from “antidepressant efficacy and side effects” survey (AESES) was reported by Li et al.^6^. The AESES survey that was also deployed to 23andMe research participants reported responses on specific drugs, and class- or drug-specific antidepressant treatment response including SSRI response, norepinephrine-dopamine reuptake inhibitor (NDRI) response^6^, citalopram/escitalopram response, SNRI response, and TRD vs. NTRD could be derived. As of this analysis, SNRI response was not previously conducted using data from AESES; we have now included this analysis in the current study. Overlapping phenotypes from AES and AESES were also meta-analyzed to increase the study power.

## Methods

### Cohorts

#### “Antidepressant Efficacy” survey (AES) cohort^11^

Saliva samples for genetic testing from approximately 56,000 research participants from 23andMe were collected under the protocol approved by Ethical and Independent Review Services^12^, a private institutional review board (IRB). Informed consent was obtained. Participants answered the AES and the ‘Your Profile and Health History’ survey online between August 2015 and January 2017.

#### “Antidepressant Efficacy and Side Effects” survey (AESES)^6^ cohort

Approximately 48,000 23andMe research participants (including the overlap with participants who took the AES) provided saliva samples and informed consent for genetic testing under the same IRB-approved protocol and answered the AESES and the “Your Profile and Health History” survey online between June 2013 and June 2015. The GWAS using data from AESES has been previously reported^6^.

#### Sample genotyping and SNP data imputation

DNA extraction and genotyping were performed, as described previosuly^6,13^. Briefly, samples were genotyped on platform variants (V1 and V2) of the Illumina HumanHap550 + BeadChip (Illumina Inc., San Diego, CA), and included ~ 25,000 custom single nucleotide polymorphisms (SNPs) selected by 23andMe, with a total of ~ 560,000 SNPs. A custom content platform (V3) based on the Illumina OmniExpress + BeadChip was used to improve the overlap, with a total of ~ 950,000 SNPs. A fully custom array platform (V4) was used which included a subset of SNPs with additional coverage of lower-frequency coding variation, and ~ 570,000 SNPs. The samples that failed to reach 98.5% call rate were re-analyzed. Prior to imputation of genotype data against the September 2013 release of 1000 Genomes^14^ Phase1 reference haplotypes, we excluded SNPs with Hardy–Weinberg equilibrium *p* < 10^−20^, call rate < 95%, or with large allele frequency discrepancies compared to European 1000 Genomes reference data^15^. Additional details on the imputation procedure are provided in Supplementary Text S1.

### Phenotype

#### Data and phenotypic analysis groups

The AES taken by 23andMe participants was designed by Janssen in collaboration with Dr. Ronald Kessler, Harvard University. The survey asked respondents about their use of antidepressants and antipsychotics over the last 5 years and the perceived qualitative effect from the treatment of the current depressive episode overall. If a study participant also used non-pharmacotherapy options, the survey attempted to tease out the contribution of pharmacotherapy (See Supplementary Fig. S1A for example questions). The list of drugs included SSRIs citalopram, escitalopram, fluoxetine, paroxetine, and sertraline; SNRIs duloxetine, venlafaxine, desvenlafaxine, and levomilnacipran; NDRI bupropion; serotonin antagonist and reuptake inhibitor trazodone; atypical antipsychotics (quetiapine, olanzapine, and aripiprazole); and serotonin modulators (vortioxetine and vilazodone), and Symbyax^®^ (a combination of olanzapine and fluoxetine).

Using phenotype data collected from the AES^11^ and genotype data from 23andMe participants, genome-wide association analyses were performed on 4 groups of phenotypes (a) NTRD (*n* = 17,214) vs. TRD (*n* = 3168), (b) SSRI responders (*n* = 8,491) vs. non-responders (*n* = 4046), (c) SNRI responders (*n* = 2055) vs. non-responders (*n* = 1950), and (d) NDRI responders (*n* = 1616) vs. non-responders (*n* = 2068). All participants included in these analyses self-reported taking antidepressants for depression. In the AES, a participant was classified as having TRD if (1) he or she took at least two antidepressants for ≥ 5–6 weeks; and (2) the overall treatment effect was not “helpful or very helpful”, or medication did not help despite the overall treatment effect was “helpful or very helpful”. A survey participant was classified as NTRD if (1) he or she only received antidepressant pharmacotherapy and the treatment effect was helpful or very helpful; (2) he or she also received non-pharmacotherapy but stated that the overall treatment effect was helpful or very helpful and medication was the main reason the treatment was helpful, or medication was important but not the main reason the treatment was helpful. In both cases, the participant took ≤ 2 antidepressant medications for more than 3–4 weeks.

A schematic flow diagram on both TRD/NTRD and class-specific responders/non-responders phenotype classification based on the AES questionnaire is provided in Supplementary Fig. S1B. Since the AES survey did not ask questions on response for each antidepressant, only participants responding to mono-pharmacotherapy were considered for class-specific responder analysis.

Using phenotype data collected from 23andMe surveys (AESES and “Your Profile and Health History”) and genotype data from 23andMe’s research participants, genome-wide association analyses were performed on one additional phenotype that was not previously analyzed^6^, SNRI responders (*n* = 2547) vs. non-responders (*n* = 1567). The responder status was defined in accordance with the previous report^6^ and described in Supplementary Text S2 and depicted in Supplemental Fig. S1C.

For each of the four AES phenotype groups, responders vs. non-responder analyses were performed both with or without AESES overlapping participants included. In addition, the responder subgroups (e.g., the resistant/non-responder groups and the non-resistant/responder groups) were also compared to healthy controls (n ~ 354,000) self-reported to be free of any of the following conditions based on the survey data captured from the “Your Profile and Health History” survey: attention-deficit/hyperactivity disorder, anxiety, schizophrenia, depression, bipolar, OCD, autism, PTSD, and insomnia as a way to confirm if the study population was similar to clinically ascertained cohorts.

#### Genome-wide association analysis

Overall analysis flow is depicted in Supplemental Fig. 1D. Specially, genome-wide analysis was restricted to a set of unrelated individuals who had > 97% European ancestry, as determined through an analysis of local ancestry. Standard quality control on directly genotyped markers excluded (1) SNPs that were only genotyped on the V1 and/or V2 platforms due to small sample size, and SNPs on chrM or chrY; (2) SNPs that failed a test for parent-offspring transmission using trio data; (3) Hardy–Weinberg *P* < 10^−20^ in Europeans; (4) SNPs with call rate of < 90%; (5) SNPs with genotyping batch effect. Imputed markers were excluded if overall *r*^2^ < 0.5, or *r*^2^ < 0.3 in any imputation batch, or with a significant imputation batch effect. For case control comparisons, association test results were computed by logistic regression assuming additive allelic effects using custom scripts implemented by 23andMe in the C^++^ programing language, which were also used to compute association test results in previous publications^6,13,16–21^. For tests using imputed data, the imputed dosages rather than best-guess genotypes were computed. Covariates for age, gender, genotype platforms, and the top five principal components to account for residual population structure were included. The association test *p*-value reported was computed using a likelihood ratio test. A *p*-value threshold of 5 × 10^−8^ was considered to be genome-wide significant^22^. No additional multiple testing correction was applied for considering multiple phenotype groups. Additional details on the method can be found in Supplementary Text S1.

#### Meta-analysis

For overlapping phenotypes between a similar analysis based on the AESES conducted previously^6^ or reported herein, and the AES study^11^ reported herein, a meta-analysis was performed. The overlapping participants who responded to both surveys were removed and only the non-overlapping participants were included in the ‘Antidepressant Efficacy’ cohort for the meta-analysis. Dosage association statistics were used in meta-analysis using PLINK^23^ (version 1.07) and fixed-effects model *p*-value is reported. Conventional genome-wide significance threshold of 5 × 10^−8^ was used to declare study-wide significance. A list of variants with an unadjusted *p*-value < 5 × 10^−4^ is also reported. In addition, meta-analyses using the AES GWAS summary statistics before removing overlapping participants and using METACARPA^24^ (a method accounting for sample overlap) were also applied and *p*_wald_, *p*_corrected_, and *p*_stouffer_ were reported. Some of the Manhattan, Q-Q and circos plots were generated using FUMA^25^, while regional plots were generated using LocusZoom v1.2^26^.

#### Genetic heritability estimates

Psychiatric Genomics Consortium (PGC) disease susceptibility summary association statistics for MDD, bipolar, and schizophrenia^27–30^ were downloaded from the PGC website (http://www.med.unc.edu/pgc/downloads) and included with the summary statistics from this study as reference datasets for genetic heritability estimates. Phenotypic variance explained by variants (both genotyped and imputed, mostly SNPs) (h^2^) for each of the phenotype groups was estimated using association statistics as implemented in LD Score regression^31^. We additionally calculated the h^2^ for the response phenotype using the genome-wide complex trait analysis (GCTA)^25^ (using pruned genotyped SNPs only) due to computation intensive step of the genetic relationship matrix (GRM) calculation.

#### Multi-marker analysis of genomic annotation (MAGMA) gene, gene-set, and cell type analysis

In addition to single-marker-based GWAS, gene and gene-set analyses were computed using MAGMA^32^ based on GWAS summary statistics. SNPs were mapped to 18,927 protein coding genes. Genome-wide significance was defined at *p* = 0.05/18,927 = 2.64 × 10^−6^. MAGMA gene-set analysis was performed for curated gene sets and GO terms obtained from the Molecular Signatures Database^33^ (MsigDB) (total of 10,894 gene sets). Lastly, MAGMA gene-property analysis was performed to test cell type specificity of phenotype using GWAS summary statistics. All MAGMA analyses were performed using FUMA^34^.

#### Annotation of variants

The implication of a causal gene for a genetic association (e.g., linking a variant to a gene) in general is not straightforward unless the variant itself causes a deleterious functional consequence. Variant-to-gene mappings (position-based, expression quantitative trait loci [eQTL]-based, or chromatin interaction-based) were generated using FUMA. eQTL-based and chromatin interaction-based mapping were used to aid the interpretation of variants identified. FUMA advocates taking position-based, eQTL, and 3-D chromatin interaction as ways to link variants to genes^34^. Open Target Platform^35^ also leverages protein quantitative trait locus (pQTL), distance to transcriptional start site (TSS) etc. The data sources for overlapping approaches (such as eQTL) are not entirely identical between bioinformatics resources such as FUMA or Open Target Genetics and therefore it is beneficial to utilize multiple tools. Open Target was used to provide additional information to aid the variant-gene linking interpretation.

#### Replication of published antidepressant treatment response GWAS top hits

Two published antidepressant treatment response GWAS meta-analyses^8,9^ have a full list of top hits with *p* < 0.0001 and *p* < 5 × 10^−6^, respectively, in the supplemental material. Despite the phenotype ascertainment difference, we attempted to replicate the findings reported focusing on the remission status endpoint and adjusting for the number of top hits in the published GWAS meta-analyses. Association passing multiple testing correction threshold was considered to be replicated; others with p < 0.05 were considered as suggestive only. Results from other treatment response endpoints were cross checked as well. No multiple testing correction was applied for 4 treatment response phenotypes that we consider in this study or multiple endpoint definitions (symptom improvement vs. remission, 2 weeks vs. 12 weeks, whole samples vs. SSRI samples only).

#### Cross reference of UK Biobank (UKB) phenome-wide association study (PheWAS) and other antidepressant treatment response results for genome wide significant variants from this study

Results from UKB PheWAS analysis performed by the Neale Lab (Broad Institute of MIT and Harvard, Cambridge, Massachusetts) are available from Open Targets. UKB PheWAS association results were assessed for top hits from the current study, especially for traits related to psychiatric conditions as corroborating evidence. An association passing phenome-wide significance threshold (*p* < 0.05/2000~ 2.5 × 10^−5^) was considered as significant, while *p* < 0.05 was considered as suggestive. Furthermore, antidepressant studies especially the STRA*D-GENDEP-MARS meta-analysis^8^ (Pharmacogenetics – PhaCoGe in https://data.broadinstitute.org/mpg/ricopili/), were assessed using SNPs in linkage disequilibrium (LD) with the genome-wide significant variants.

## Results

The sample size and demographics for each phenotype definition derived from AES are described in Table 1 with additional details in Supplementary Table S1.

**Table 1:** Sample size and basic demographic and genomic control inflation factor.

Study	Group	Total	Gender		Age	Platform						Genomic control inflation factor		
			M	F	(0,30)	(30,45)	(45,60)	z	v1/v2	v3	v4	λ	λ_1000_	λ_10000_
AES	*SSRI responders vs. non-responders*													
	Responders	8491	2048	6443	993	2103	2511	2884	49	638	7804			
	Non-responders	4046	1117	2929	728	1189	1171	958	18	312	3716	1.015	1.003	1.027
AES	*SNRI responders vs. non-responders*													
	Responders	2055	451	1604	111	443	747	754	5	176	1874			
	Non-responders	1950	504	1446	195	544	664	547	5	172	1773	1.017	1.008	1.083
AES	*NDRI responders vs. non-responders*													
	Responders	1616	502	1114	148	455	567	446	10	124	1482			
	Non-responders	2068	602	1466	280	621	679	488	9	183	1876	1.01	1.006	1.058
AES	*NTRD vs. TRD*													
	NTRD	17214	4281	12933	1733	4182	5501	5798	95	1323	15796			
	TRD	3168	894	2274	493	893	1002	780	14	265	2889	1.016	1.003	1.031
*AESES*	*SNRI responders vs. non-responders*													
	Responders	2547	656	1891	160	614	833	940	37	761	1749	1.013	1.007	1.07
	Non-responders	1567	508	1059	159	491	510	407	18	517	1032			
*AESES*	*NDRI responders vs. non-responders*													
	*Responders*	*2675*	*799*	*1876*	*297*	*710*	*914*	*754*	*46*	*840*	*1789*			
	*Non-responders*	*1861*	*656*	*1205*	*300*	*569*	*541*	*451*	*27*	*663*	*1171*	*1.007*	*1.003*	*1.032*
*AESES*	*SSRI responders vs. non-responders*													
	*Responders*	*6348*	*1770*	*4578*	*671*	*1543*	*2033*	*2101*	*103*	*1997*	*4248*			
	*Non-responders*	*3340*	*1229*	*2111*	*666*	*1106*	*863*	*705*	*44*	*1128*	*2168*	*1.008*	*1.002*	*1.018*
*AESES*	*NTRD vs. TRD*													
	*Responders*	*7795*	*2204*	*5591*	*799*	*1834*	*2547*	*2615*	*122*	*2321*	*5352*			
	*Non-responders*	*1311*	*517*	*794*	*271*	*420*	*349*	*271*	*14*	*445*	*852*	*1.019*	*1.009*	*1.086*

*AES* Antidepressant Efficacy Survey, *AESES* Antidepressant Efficacy and Side Effects Survey, *NDRI* norepinephrine–dopamine reuptake inhibitor, *NTRD* non-treatment-resistant depression, *SNRI* serotonin-norepinephrine reuptake inhibitor, *SSRI* selective serotonin reuptake inhibitor, *TRD* treatment-resistant depression. Note that analyses reported previously in Li et al., 2016 have been *italicized*.

### SSRI responders vs. non-responders: rs4884091

**Fig. 1:**
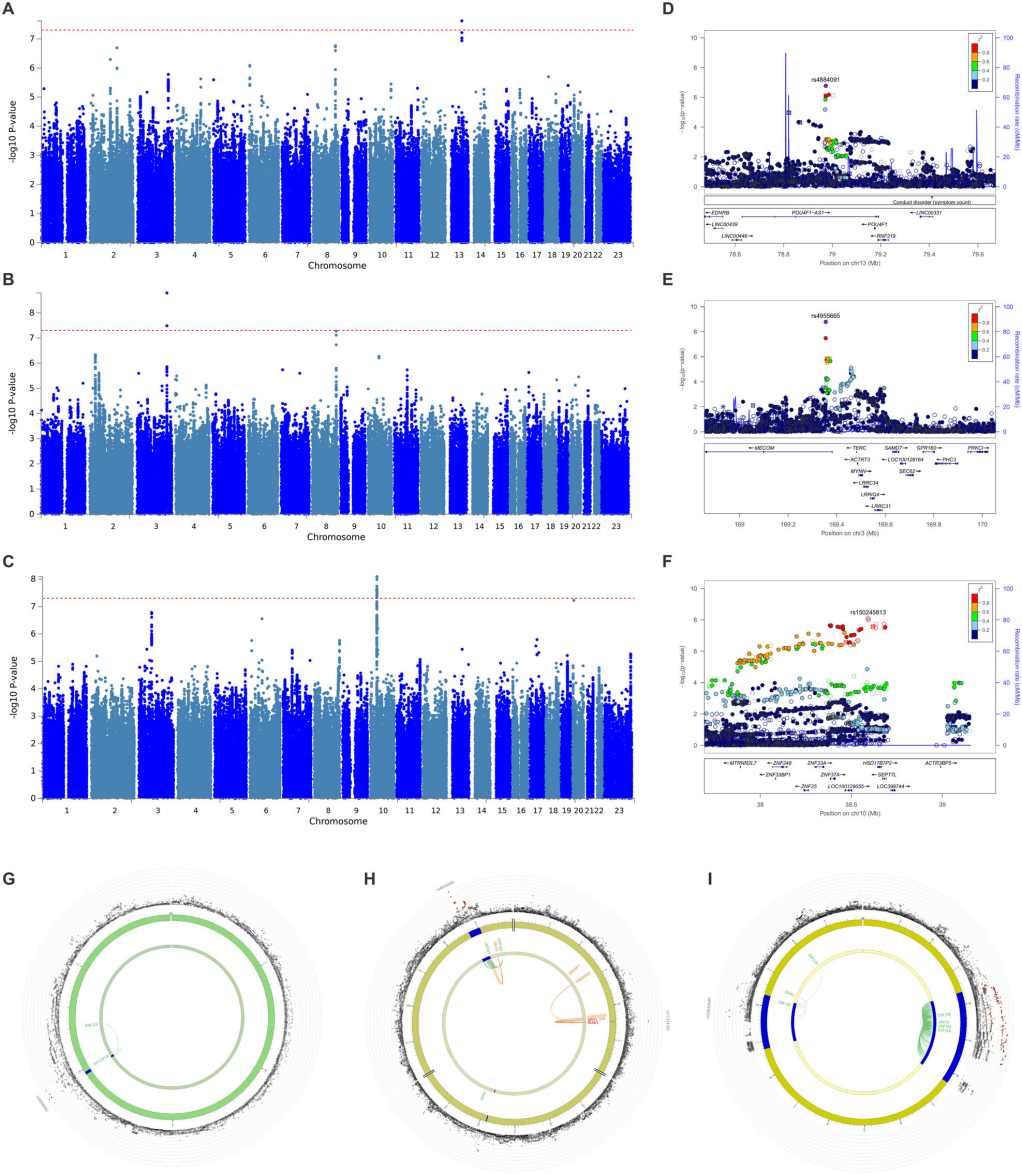
Genome-wide significant association signals. (A) Manhattan plots for SSRI GWAS in AES cohort; (B) SNRI responders vs. non-responders GWAS meta-analysis; (C) NTRD vs. TRD GWAS meta-analysis; (D) Regional plot for chromosome 13; (E) Regional plot for chromosome 3; (F) Regional plot for chromosome 10; (G) Circos plot for chromosomes 13; (H) Circos plot for chromosomes 3; (I) Circos plot for chromosomes 10. The dotted line indicates genome-wide significance threshold of 5 × 10^−8^. For the regional association plot generated by LocusZoom^26^, SNPs in genomic risk loci are color-coded as a function of their *r*^2^ to the index SNP in the locus, as follows: red (*r*^2^ > 0.8), orange (*r*^2^ > 0.6), green (*r*^2^ > 0.4) and light blue (*r*^2^ > 0.2). SNPs that are not in LD with the index SNP (with *r*^2^ ≤ 0.2) are dark blue, while SNPs with missing LD information are shown in gray. For the circos plot, the outer most layer is Manhattan plot and the middle layer highlights genomic risk loci (as defined by FUMA using minimum P-value of lead SNPs of 1 × 10^-5^ and default values for other parameters) in blue in, while the inner most layer highlights eQTLs and/or chromatin interactions. Only SNPs with *p* < 0.05 are displayed in the outer ring. SNPs in genomic risk loci are color-coded as a function of their maximum r^2^ to the one of the independent significant SNPs in the locus, as follows: red (*r*^2^ > 0.8), orange (*r*^2^ > 0.6), green (*r*^2^ > 0.4) and blue (*r*^2^ > 0.2). SNPs that are not in LD with any of the independent significant SNPs (with *r*^2^ ≤ 0.2) are gray. The rsID of the top SNPs in each risk locus are displayed in the most outer layer. For the inner most layer, if the gene is mapped only by chromatin interactions or only by eQTLs, it is colored orange or green, respectively. It is colored red when the gene is mapped by both. *AES* Antidepressant Efficacy Survey, *GWAS* genome-wide association analysis, *SSRI* selective serotonin reuptake inhibitor, *SNRI* serotonin-norepinephrine reuptake inhibitor, *TRD* treatment-resistant depression.

In the SSRI GWAS (SSRI responders vs. non-responders), one genomic region (index SNP rs4884091, *p* = 2.42 × 10^−8^, OR = 1.21) was identified (Fig. 1A, D, Supplementary Fig. S2A) in *RNF219-AS1* (also known as OBI1 antisense RNA 1 [*OBI-AS1*]) between endothelin receptor type B (*EDNRB*) and POU class 4 homeobox 1 (*POU4F1*) passing the genome-wide significance threshold (*p* < 5 × 10^−8^) in the AES cohort (Table 2). The lead SNP rs4884091 is an eQTL variant (*p*_eQTL_ = 8.19 × 10^−28^ in eQTLGen) for ring finger protein 219 (*RNF219*) (Fig. 1G). The lead SNP rs4884091 is also an eQTL that regulates ribosomal protein L31 pseudogene 54 (*RPL31P54*) expression level in the nucleus accumbens (*p*_eQTL_ = 1.6 × 10^−10^)^36^. However, the functional consequence of *RPL31P54* is unclear. *RNF219* is ubiquitously expressed thus making it a less attractive candidate as a relevant gene^37^. Unlike other genes implicated in this study, *RPL31P54* is exclusively expressed in the brain (Supplementary Fig. S3). In the SNRI GWAS (SNRI responders vs. non-responders), no variant passed genome-wide significance (Supplementary Figs. S2B, S4A). Nor were there any variants passing the genome-wide significance level in the NDRI GWAS (NDRI responders vs. non-responders) (Supplementary Figs. S2C and S4B), or in the TRD GWAS (NTRD vs. TRD) (Supplementary Figs. S2D and S4C). SNRI GWAS in the AESES cohort alone also did not yield any genome-wide significant hits (Supplementary Figs. S2E and S4D). Suggestive association signals found in these analyses are described in Supplemental Text S3.

**Table 2:** Genome-wide significant SNPs for each phenotype in either AES cohort or in meta-analysis.

SNP	CHR	BP	A1	A2	AES						AESES				Meta-analysis					N	Annotation	Distance	Gene Symbol	Gene Context
					*p*^*a*^	OR^a^	FRQ^b^	*p*^*b*^	OR^b^	SE^b^	FRQ	*p*	OR	SE	*p*_*Fixed_effect*_	OR	*p*_wald_	*p*_corrected_	*p*_stouffer_					
*SSRI responders vs. non-responders*																								
rs4884091	13	78,973,223	G	A	**2.42** × **10**^**−08**^	1.21	0.79	3.49 × 10^−07^	1.22	0.04	0.80	0.02	1.09	0.04	1.67 × 10^−07^	1.16	**1.21** × **10**^**−08**^	**1.37** × **10**^**−08**^	**1.34** × **10**^**−08**^	22225	ncRNA_intronic	0	*RNF219-AS1*	*EDNRB---[]---POU4F1*
*SNRI responders vs. non-responders*																								
rs4955665	3	169,355,019	T	C	3.13 × 10^−04^	1.19	0.59	2.64 × 10^−04^	1.21	0.05	0.60	1.21E-06	1.27	0.05	**1.62** × **10**^**−09**^	1.25	**4.35** × **10**^**−09**^	**3.94** × **10**^**−09**^	**4.04** × **10**^**−09**^	8119	intronic	0	*MECOM*	*[MECOM]*
*NTRD vs. TRD*																								
rs150245813	10	38,592,780	T	G	7.32 × 10^−08^	0.79	0.88	7.35 × 10^−07^	0.79	0.05	0.88	1.40E-03	0.81	0.07	**8.07** × **10**^**−09**^	0.80	**8.88** × **10**^**−10**^	**4.22** × **10**^**−10**^	**4.08** × **10**^**−10**^	29488	ncRNA_intronic	0	*ENSG00000226113*	*ZNF37A---[]---LINC00999*

*AES* Antidepressant Efficacy Survey, *AESES* Antidepressant Efficacy and Side Effects Survey, *BP* base-pair, *CHR* chromosome, *FRQ* frequency, *NDRI* norepinephrine-dopamine reuptake inhibitor, *NTRD* non-treatment-resistant depression, *OR* odds ratio, *SE* standard error, *SNP* single nucleotide polymorphism, *SNRI* serotonin-norepinephrine reuptake inhibitor, *SSRI* selective serotonin reuptake inhibitor, *TRD* treatment-resistant depression.

^a^with overlapping samples between AES and AESES cohorts.

^b^without overlapping samples between AES and AESES cohorts.

*p*_Fixed_effect_ was computed between the AES (without overlapping samples) and AESES cohorts using PLINK, while *p*_wald_, *p*_corrected_, and *p*_stouffer_ were computed between AES (with overlapping samples) and AESES cohorts using METACARPA.

Bold numbers indicate *p* < 5 × 10^−8^.

In the meta-analysis for SSRI response, the association signal for rs4884091 was strengthened (*p* from 3.49 × 10^−7^ to 1.67 × 10^−7^ in the PLINK analysis without overlapping samples or from 2.42 × 10^−8^ to 1.21 × 10^−8^ in the METACARPA analysis with overlapping samples) (Table 2, Supplementary Figs. S2F and S4E).

### SNRI responder vs. non-responder meta-analysis: rs4955665

Meta-analysis for SNRI response phenotype identified a genomic region (lead SNP rs4955665, *p* = 1.62 × 10^−9^, OR = 1.25 as calculated by PLINK fixed effect model; *p*_wald_ = 4.35 × 10^−9^, *p*_corrected_ = 3.94 × 10^−9^, and *p*_stouffer_ = 4.04 × 10^−9^ as calculated by METACARPA) in the intronic region of MDS1 and EVI1 complex locus (*MECOM*) in chromosome 3 passing the genome-wide significance threshold (Fig. 1B and E, Supplementary Fig. S2G). The lead SNP is an eQTL variant (*p*_eQTL_ = 1.26 × 10^−8^) for G protein-coupled receptor 160 (*GPR160*) in eQTLGen (blood eQTL data)^38^ and leucine rich repeats and IQ motif containing 4 (*LRRIQ4*, brain tissues) (Fig. 1H). There is also evidence of intra-chromosomal interaction between this associated genomic region and the region including SEC62 homolog, preprotein translocation factor (*SEC62*) (Fig. 1H). The associations for the SNRI response phenotype had comparable effect size between the two studies with a stronger association in the previous AESES study (*p* = 1.21 × 10^-6^, OR = 1.27) than the current AES study (*p* = 2.64 × 10^−4^, OR = 1.21).

No variant passed genome-wide significance in NDRI GWAS meta-analysis (Supplementary Figs. S2H and S4F).

### NTRD vs. TRD meta-analysis: rs150245813

Meta-analysis for TRD phenotype derived from the AESES and the AES identified one additional genomic region (lead SNP rs150245813, *p* = 8.07 × 10^−9^, OR = 0.80 as calculated by PLINK fixed effect model; *p*_wald_ = 8.88 × 10^−10^, *p*_corrected_ = 4.22 × 10^−10^, and *p*_stouffer_ = 4.08 × 10^−10^ as calculated by METACARPA) in 10p11.1 passing genome-wide significance threshold (Fig. 1C, Supplementary Figs. S2I). The associations had comparable effect size between the two studies with a stronger association in the current AES study (*p* = 7.35 × 10^−7^, OR = 0.79) than the previous AESES study (*p* = 1.40 × 10^−3^, OR = 0.81). The associated locus spans across multiple genes (Fig. 1F) and harbors eQTL variants (Fig. 1I). The lead SNP rs150245813 is an eQTL variant (Data source: the Genotype-Tissue Expression (GTEx) project^39,40^ Analysis Release V7) for zinc finger protein 48 (*ZNF48*) (*p*_eQTL_ = 3.3 × 10^−12^ in colon—Sigmoid and *p*_eQTL_ = 0.007 in brain—hippocampus), RP11-672F9.1 (*p*_eQTL_ = 2.6 × 10^−20^ in testis), and RP11-258F22.1 (*p*_eQTL_ = 2.4 × 10^−3^ in brain—frontal cortex [BA9] and *p*_eQTL_ = 2.6 × 10^−6^ in colon—sigmoid). On the other hand, rs2505705, a SNP in LD with the lead SNP (r^2 = 0.66) is an eQTL variant for zinc finger protein (*ZNF33A*) (*p*_eQTL_ = 1 × 10^−9^) and zinc finger protein 25 (*ZNF25*) (*p*_eQTL_ = 1 × 10^−9^) in CommonMind Consortium^41^ (CMC), and for *ZNF248* (*p*_eQTL_ = 7.65 × 10^−18^), zinc finger protein 25 (*ZNF25*) (*p*_eQTL_ = 2.87 × 10^−58^) and zinc finger protein 37 A (*ZNF37A*) (*p*_eQTL_ = 2.34 × 10^−11^) in eQTL Gen^38^.

A full list of suggestive association with *p* < 5 × 10^−4^ for all four treatment response endpoints is available in the Supplemental Table S2.

Analysis of the heritability estimates for responders vs. non-responders is shown in Supplementary Table S3. Overall, the heritability estimates for response phenotypes are still unreliable with confidence intervals crossing zero except two that were estimated using GCTA, suggesting the sample size is still not sufficiently large to yield a reliable estimate. Most of the disease phenotypes (responders vs. controls, or non-responders vs. controls) were similar to those estimated for MDD cases vs. controls from PGC, as observed in the AESES study^6^.

MAGMA gene analysis identified one and three genes passing the multiple testing correction threshold for SNRI and TRD phenotypes, respectively, in the AES cohort (Table 3, Supplementary Table S4), including lymphotoxin beta (*LTB*), an inflammation-related gene implicated to be associated with TRD^42^. None of the gene-level MAGMA associations (using meta-analysis association statistics) yield genome-wide significance (Supplementary Table S5).

**Table 3:** Genome-wide significant findings for MAGMA gene-based analysis.

Gene	CHR	START	STOP	N	ZSTAT	*p*	SYMBOL
*AES cohort*							
*SNRI responders vs. non-responders*							
ENSG00000154736	21	28,280,231	28,348,832	4005	4.6289	1.84 × 10^−06^	*ADAMTS5*
*NTRD vs. TRD*							
ENSG00000227507	6	31,538,302	31,560,299	20382	4.6228	1.89 × 10^−06^	*LTB*
ENSG00000204482	6	31,543,901	31,566,686	20382	4.7884	8.40 × 10^−07^	*LST1*
ENSG00000204475	6	31,546,672	31,570,762	20382	4.799	7.97 × 10^−07^	*NCR3*

*AES* Antidepressant Efficacy Survey, *CHR* chromosome, *MAGMA* Multi-marker Analysis of GenoMic Annotation, *NTRD* non-treatment-resistant depression, *SNRI* serotonin-norepinephrine reuptake inhibitors, *TRD* treatment-resistant depression.

Only suggestive enrichment was observed for MAGMA gene-set analysis across all phenotypes (Supplementary Tables S6 and S7). NDRI analysis in AES cohort suggested an enrichment of genes involved in organ or tissue specific immune response (*p* = 5 × 10^−5^) and innate immune response (*p* = 2.9 × 10^−4^) (Supplementary Table S6). SNRI analysis in AESES cohort also suggested an enrichment of genes involved in inflammatory response (*p* = 9.75 × 10^−5^, Supplementary Table S6) and inflammasome complex (*p* = 0.0008). Meta-analysis suggested an enrichment of genes involved in interleukin signaling (*p* = 8.42 × 10^*-*5^) in SSRI treatment response (Supplementary Table S7).

Cell type analysis of SNRI METACARPA meta-analysis results revealed potential enrichment of GABAergic neurons (*p* = 0.03, *p*_adj_ = 0.08 when adjusted within Allen Brain Atlas Cell Type human MTG^43^ dataset and multiple single cell RNA-Seq [scRNA-Seq] datasets showed trends towards GABAergic neurons), while SSRI, NDRI, and TRD meta-analysis results revealed potential enrichment of glutamatergic neurons and microglia (Supplementary Table S8). The enrichment was not statistically significant when adjusted for across all scRNA-Seq datasets tested.

We used our study results to replicate antidepressant treatment response outcome reported in the literature^8,9^. Among the top hits (*p* < 0.0001) from remission after up to 12 weeks of treatment in the meta-analysis of SSRI-treated participants in GENDEP and STAR*D (top hits *n* = 54) and the meta-analysis of the entire GENDEP, MARS, and STAR*D samples (top hits *n* = 60), only rs6540437 near complement C3b/C4b receptor 1 like (*CR1L*) that was suggestively associated with SSRI remission (*p* = 0.00004 in GENDEP and STAR*D meta-analysis) was replicated (*p* = 0.0008 for NTRD vs. TRD analysis) in this study (*p* < = 0.05/54~ 0.0009) with consistent directional effect. The results from NDRI responders vs. non-responder (*p* = 0.003) and SSRI responders vs. nonGenome-responders (*p* = 0.01) were suggestively supportive. Other replication results of suggestive associations are shown in Supplementary Table S9 and S10.

Among the genome-wide significant variants identified by Fabbri et al.^9^, in the re-analysis of GENDEP and STAR*D samples, rs76191705 had a nominal association in the NDRI responder analysis in AESES cohort (*p* = 0.02, OR = 1.58) but not in the AES cohort (*p* = 0.08, OR = 0.70) nor in the SSRI responder analyses. rs116692768 had a nominal association in the NDRI responder analysis in AESES cohort (*p* = 0.01, OR = 0.71) but the directionality was opposite and there was no association in the AES cohort or in the SSRI analysis in either cohort. Furthermore, Wei et al. identified a putative functional variant rs7905446 in serotonin receptor 7 (*HTR7*) that regulates transcript activity and transcription factor binding with the rare variant carrier associated with better response in GENDEP and MARS, as well as two treatment arms in a bipolar cohort^44^. This finding was replicated in the citalopram/escitalopram analysis (*p* = 0.04, OR = 1.1) in the AESES cohort^6^.

Conversely, we examined corroborating evidence from UKB PheWAS and other antidepressant treatment response studies for the genome-wide significant variants identified in the current study. Interestingly, rs4884091 that was associated with SSRI responders (vs. non-responders) was also suggestively associated with “manic/hyper symptoms: I was more creative or had more ideas than usual” (*p* = 0.003) in UKB PheWAS. In the meta-analysis between AES and AESES cohorts, we identified two additional genome-wide significant loci. The rs4955665 variant associated with SNRI response was also suggestively associated with “longest period of un-enthusiasm / disinterest” (*p* = 0.0004), “manic/hyper symptoms: I was more talkative than usual” (*p* = 0.02), and “diagnoses - main ICD10: F99 Mental disorder, not otherwise specified” (*p* = 0.004) in UKB (data source: Open Target, PheWAS analysis performed by the Neale Lab)^35^. The rs150245813 variant associated with NTRD (vs. TRD) was also additionally suggestive of association with “Diagnoses - main ICD10: F33 Recurrent depressive disorder” (*p* = 0.01) and “Diagnoses - main ICD10: F43 Reaction to severe stress, and adjustment disorders” (*p* = 0.02). However, none of the PheWAS-suggestive associations would be significant after adjusting for more than 2,000 traits tested. In addition, the genome-wide significant variants from this study were not replicated in the STAR*D-MARS-GENDEP meta-analysis^8^ for remission status after up to 12 weeks of treatment. Specifically, rs2804669 in LD (*r*^2^ = 0.5, D’ = 1) with rs150245813 was not associated with remission status (*p* = 0.88). Likewise, rs4955666 in LD (*r*^2^ = 0.90, D’ = 1) with rs4955665 was not associated with remission (*p* = 0.68). Lastly, rs9318544 in LD (*r*^2^ = 0.92, D’ = 0.98) with rs4884091 was not associated with remission (*p* = 0.64).

## Discussion

In the current analysis, the use of GWAS identified several genetic markers potentially associated with TRD and with antidepressant treatment response in a large population of individuals using self-reported outcomes. To the best of our knowledge, this study included the largest cohort to date for evaluation of GWAS of antidepressant efficacy.

Among the variants, genes, and gene sets that were identified in various analyses, a common theme on immune regulation emerges. *LTB* is one of the genes implicated to be associated with TRD in gene-based MAGMA analysis. *LTB* is an inducer of the inflammatory response system, implicating the role of inflammatory response modulation in TRD, which is consistent with the role of inflammation in MDD^45,46^ and antidepressant treatment response^46,47^. However, it is noteworthy that there are three genes in this region of chromosome 6 associated with TRD status in the gene-base analysis. This is a gene-dense, high-LD region of chromosome 6, which raises a caveat that it could be difficult to localize the causal associations in this region. MAGMA gene-set analysis also suggested an association with genes involved in organ or tissue specific immune response and innate immune response in NDRI analysis. SNRI analysis in AESES cohort also suggested an enrichment of genes involved in inflammatory response and the inflammasome complex. Meta-analysis suggested an enrichment of genes involved in interleukin signaling in SSRI treatment response, consistent with the theme that inflammatory response plays a role in antidepressant treatment response. Replication of STAR*D and GENDEP meta-analysis also highlighted the involvement of *CR1L*. Dysregulation of synaptic plasticity and deficits in functional connectivity are hypothesized to contribute to symptoms associated with MDD. Holmes et al.^48^, used the synaptic vesicle glycoprotein 2 A (SV2A) radioligand to index the number of nerve terminals as an indirect estimate of synaptic density and showed that the severity of depressive symptoms was inversely correlated with SV2A density. In mice models of demyelinating diseases, synapse loss coincided with gliosis and increased complement component C3 at synapses. Overexpression of the complement inhibitor Crry/Cr1l at C3-bound synapses decreased microglial engulfment of synapses^49^. It is intriguing that we observed the replication of *CR1L* polymorphism for SSRI treatment response, despite the difference in phenotypes. Although the present study is suggestive of the converging theme of involvement of immune processes, several limitations are worth mentioning. First, it will be most convincing if the finding was identified in one of the cohorts (AESES or AES) and strengthened in the meta-analysis. This is likely due to combination of sample size being not large enough (i.e., small compared to disease phenotype GWAS meta-analysis) and because the phenotype definition was not based on ascertainment by depression symptom severity scales. Second, the gene set evidence is suggestive as they did not pass the stringent multiple testing correction threshold.

Despite the use of self-reported phenotypes, the genetic heritability for MDD disease phenotype estimated from our cohort is comparable to that estimated from the PGC2 MDD cohort^50^, supporting that the disease phenotype ascertained by self-reporting is comparable with that ascertained by clinical assessment. However, we cannot readily extrapolate this finding to the self-reported treatment outcome phenotype. To our knowledge, possibly due to inadequate sample size of most studies, no reliable estimates of the heritability of antidepressant treatment response have been published to-date. The Janssen-23andMe AESES survey GWAS, based on self-reported outcomes, identified one genome-wide association locus but did not take treatment duration into consideration^6^. The AES used in this study was aimed to interrogate self-report predictors of TRD and did consider antidepressant exposure time. The genome wide significant locus reported from NDRI analysis in the AESES cohort^6^ was not replicated in the AES cohort.

A limitation of this study was the treatment outcome phenotype was self-reported and that heritability estimates are still very low/unreliable with the meta-analysis sample size used for responder vs. non-responder analysis. Another limitation is that survey participants were not representative of all patients with depression, as they volunteered to provide samples for genetic testing. In addition, outcome assessments were based entirely on retrospective self-reporting. The genome significant findings reported here have not been replicated and thus require further study to provide supporting or refuting evidence.

The current study appears to be the largest cohort ever evaluated for GWAS of antidepressant efficacy. Our results identified novel associations of genetic variants with antidepressant responders vs. non-responders, but the findings require replication. Further, the meta-analysis of two antidepressant efficacy surveys identified two additional loci at the single variant level for TRD and SNRI response phenotype. Several additional loci at gene level passed genome-wide significance for both the TRD and SNRI response phenotype. Future GWAS with large sample size and meta-analysis with additional cohorts will be needed to replicate the findings reported here. Meta-analysis with other antidepressant treatment response studies may eventually have enough study power to help to predict treatment outcome to a specific antidepressant class and/or in TRD vs. NTRD.

## Supplementary information

Supplementary information

Supplementary Figure S1

Supplementary Figure S2

Supplementary Figure S3

Supplementary Figure S4

Supplementary Text

Supplementary Tables

Supplementary Table S2

Supplementary Table S10
