# Supplementary material for: Genome-wide association studies of antidepressant class response and treatment-resistant depression
Source: Transl Psychiatry. 2020 Oct 26;10:360. doi: 10.1038/s41398-020-01035-6 (PMC7589471; doi:10.1038/s41398-020-01035-6)
Supplement: Supplementary file 1 — Supplementary information [file 41398_2020_1035_MOESM1_ESM.docx]

**Supplementary Information**

**Supplementary Figure S1.** Schematic flow diagram for overall analysis and survey design and phenotype definition. (A) Selected questions from AES (B) Phenotype definition using AES (C) Selected questions from AESES and phenotype definition (D) Overall analysis flow

**Supplementary Figure S2.** Q-Q plots for GWAS (observed vs. expected quantiles for the GWAS p-values) in different cohorts (A) SSRI GWAS in AES cohort (B) SNRI GWAS in AES cohort(C) NDRI GWAS AES cohort (D) NTRD vs TRD GWAS in AES cohort (E) SNRI GWAS in AESES cohort (F) SSRI meta-analysis (G) SNRI meta-analysis (H) NDRI GWAS meta-analysis (I) NTRD vs TRD meta-analysis

*AES* Antidepressant Efficacy Survey, *GWAS* genome-wide association analysis, *NDRI* norepinephrine-dopamine reuptake inhibitor, *NTRD* non-treatment-resistant depression,

*SNRI* serotonin-norepinephrine reuptake inhibitor*, SSRI* selective serotonin reuptake inhibitor, *TRD* treatment-resistant depression

**Supplementary Figure S3.** *RPL31P54* gene expression profile from GTEx

*GTEx* Genotype-Tissue Expression

**Supplementary Figure S4.** Manhattan plots for GWAS (A) SNRI GWAS (responders vs. non-responders) in AES cohort(B) NDRI GWAS (responders vs. non-responders) in AES cohort (C) TRD GWAS (NTRD vs. TRD) in AES cohort (D) SNRI GWAS (responders vs. non-responders) in AESES cohort (E) SSRI GWAS meta-analysis (SSRI-responders vs. non-responders) (F) NDRI GWAS meta-analysis (NDRI-responders vs. non-responders)

*AES* Antidepressant Efficacy Survey, *GWAS* genome-wide association analysis, *NDRI* norepinephrine-dopamine reuptake inhibitor, *NTRD* non-treatment-resistant depression,

*SNRI* serotonin-norepinephrine reuptake inhibitor*, SSRI* selective serotonin reuptake inhibitor, *TRD* treatment-resistant depression

**Supplementary Table S1.** Sample size and basic demographic and genomic control inflation factor

**Supplementary Table S2.** Top hits from GWAS meta-analysis with *p* ≤ 5x10^-4^ together with association statistics from AESES and AES cohort and ANNOVAR annotations (a) NTRD vs. TRD; (b) SSRI responders vs. non-responders; (c) SNRI responders vs. non-responders; and (d) NDRI responders vs. non-responders

**Supplementary Table S3.** Genetic heritability estimates using LD Score Regression and Genome-wide complex trait analysis Supplementary

**Supplementary Table S4.** MAGMA gene level association analysis for AES and AESES cohorts (*p* < 0.0001)

**Supplementary Table S5.** MAGMA gene level association using meta-analysis summary association statistics

**Supplementary Table S6.** MAGMA gene set enrichment analysis in AES cohort (*p* < 0.0005)

**Supplementary Table S7.** MAGMA gene set enrichment analysis using meta-analysis summary association statistics (*p* < 0.0005)

**Supplementary Table S8.** MAGMA cell type enrichment analysis using meta-analysis summary association statistics

**Supplementary Table S9.** Replication results of the reported suggestive associations from GENDEP, MARS, and STAR*D meta-analysis study

**Supplementary Table S10.** Replication results of the reported suggestive association from Fabbri et al (GENDEP and STAR*D meta-analysis) study

**Supplementary Text S1.** Genome-wide association analysis

**Supplementary Text S2.** Description of AESES phenotype definition.

**Supplementary Text S3.** Description of top hits from genome-wide association analysis
