# Supplementary material for: Genome-wide association studies of antidepressant class response and treatment-resistant depression
Source: Transl Psychiatry. 2020 Oct 26;10:360. doi: 10.1038/s41398-020-01035-6 (PMC7589471; doi:10.1038/s41398-020-01035-6)
Supplement: Supplementary file 2 — Supplementary Figure S1 [file 41398_2020_1035_MOESM2_ESM.pptx]

## Slide 1
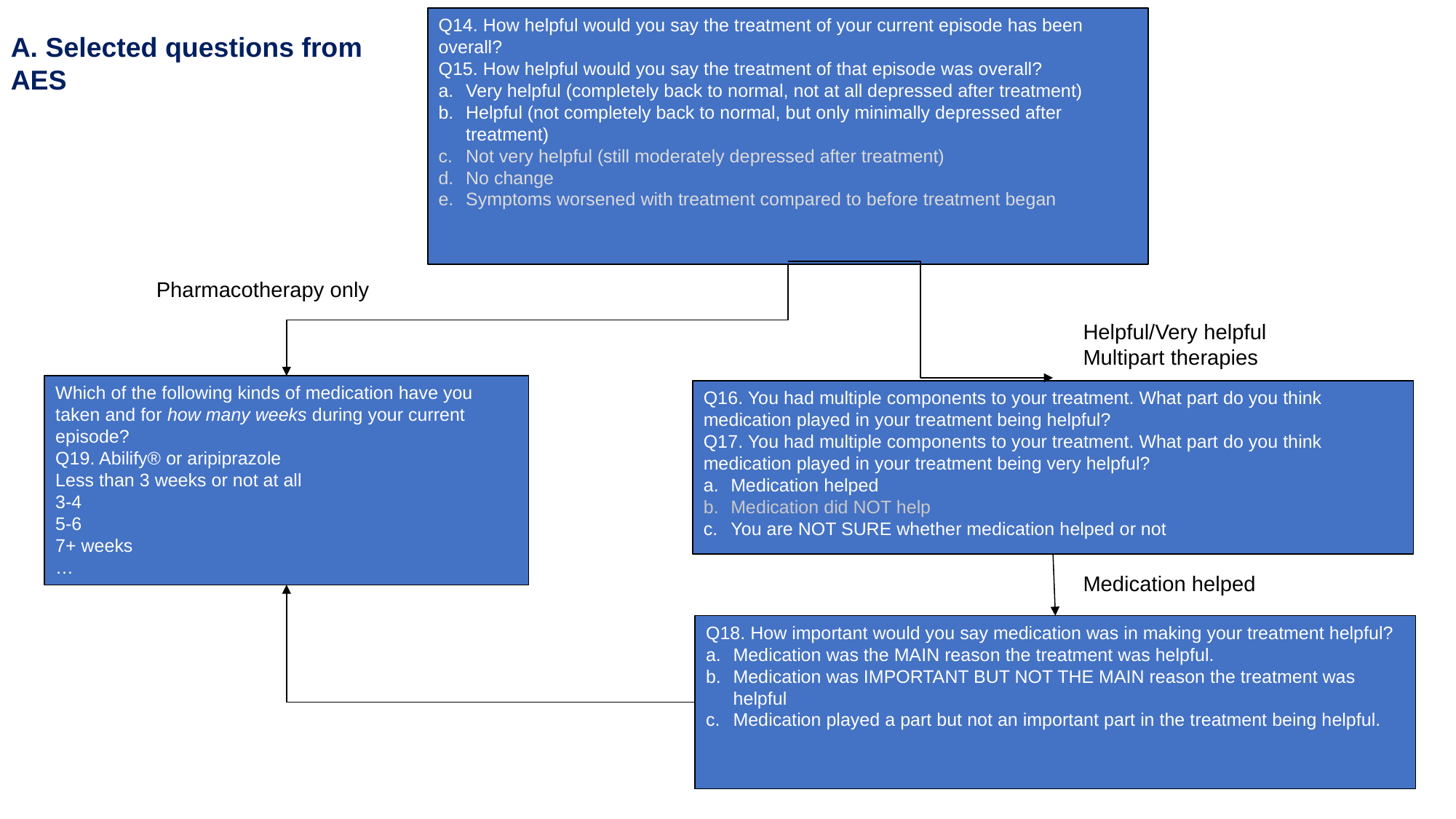

Q14. How helpful would you say the treatment of your current episode has been overall?
Q15. How helpful would you say the treatment of that episode was overall?
Very helpful (completely back to normal, not at all depressed after treatment)
Helpful (not completely back to normal, but only minimally depressed after treatment)
Not very helpful (still moderately depressed after treatment)
No change
Symptoms worsened with treatment compared to before treatment began
Pharmacotherapy only
Helpful/Very helpful
Multipart therapies
Which of the following kinds of medication have you taken and for how many weeks during your current episode?
Q19. Abilify® or aripiprazole
Less than 3 weeks or not at all
3-4
5-6
7+ weeks
…
Q16. You had multiple components to your treatment. What part do you think medication played in your treatment being helpful?
Q17. You had multiple components to your treatment. What part do you think medication played in your treatment being very helpful?
Medication helped
Medication did NOT help
You are NOT SURE whether medication helped or not
Medication helped
Q18. How important would you say medication was in making your treatment helpful?
Medication was the MAIN reason the treatment was helpful.
Medication was IMPORTANT BUT NOT THE MAIN reason the treatment was helpful
Medication played a part but not an important part in the treatment being helpful.
A. Selected questions from AES
1

## Slide 2
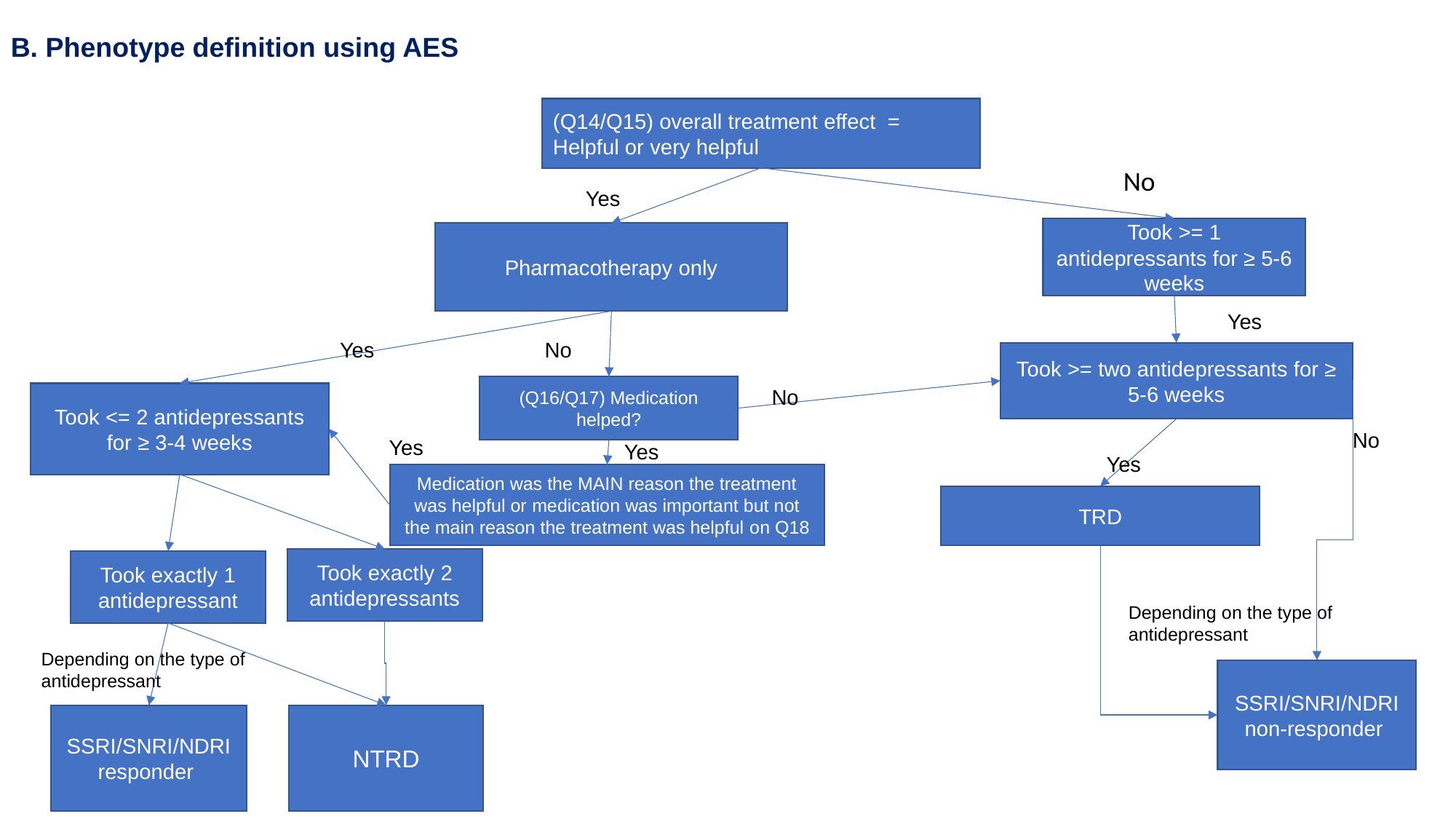

B. Phenotype definition using AES
(Q14/Q15) overall treatment effect = Helpful or very helpful
No
	Yes
Took >= 1 antidepressants for ≥ 5-6 weeks
Pharmacotherapy only
Yes
	Yes	 No
Took >= two antidepressants for ≥ 5-6 weeks
(Q16/Q17) Medication helped?
No
Took <= 2 antidepressants for ≥ 3-4 weeks
No
Yes
Yes
Yes
Medication was the MAIN reason the treatment was helpful or medication was important but not the main reason the treatment was helpful on Q18
TRD
Took exactly 2 antidepressants
Took exactly 1 antidepressant
Depending on the type of antidepressant
Depending on the type of antidepressant
SSRI/SNRI/NDRI non-responder
SSRI/SNRI/NDRI responder
NTRD

## Slide 3
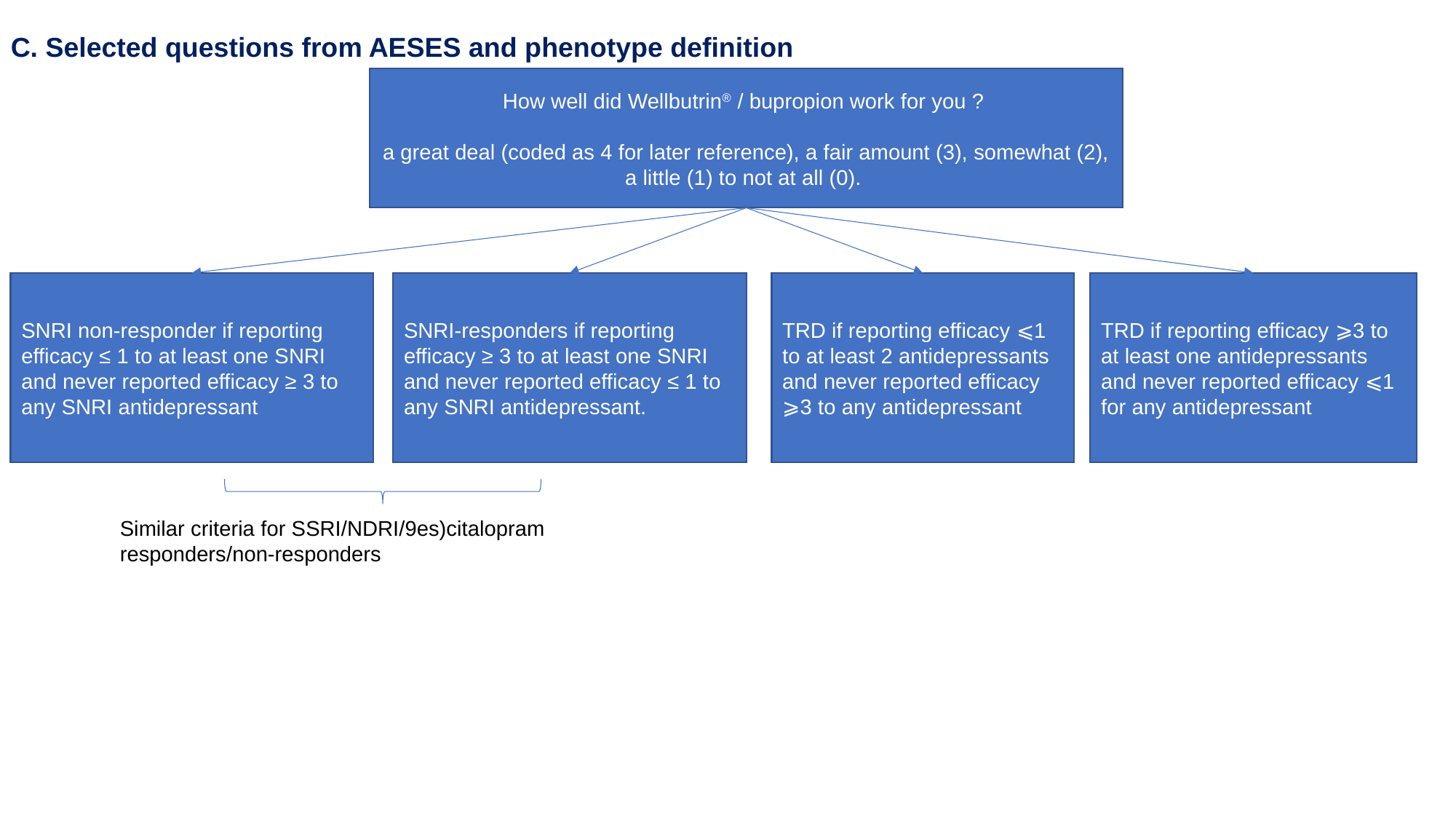

C. Selected questions from AESES and phenotype definition
How well did Wellbutrin® / bupropion work for you ?
a great deal (coded as 4 for later reference), a fair amount (3), somewhat (2), a little (1) to not at all (0).
SNRI non-responder if reporting efficacy ≤ 1 to at least one SNRI and never reported efficacy ≥ 3 to any SNRI antidepressant
SNRI-responders if reporting efficacy ≥ 3 to at least one SNRI and never reported efficacy ≤ 1 to any SNRI antidepressant.
TRD if reporting efficacy ⩽1 to at least 2 antidepressants and never reported efficacy ⩾3 to any antidepressant
TRD if reporting efficacy ⩾3 to at least one antidepressants and never reported efficacy ⩽1 for any antidepressant
Similar criteria for SSRI/NDRI/9es)citalopram responders/non-responders

## Slide 4
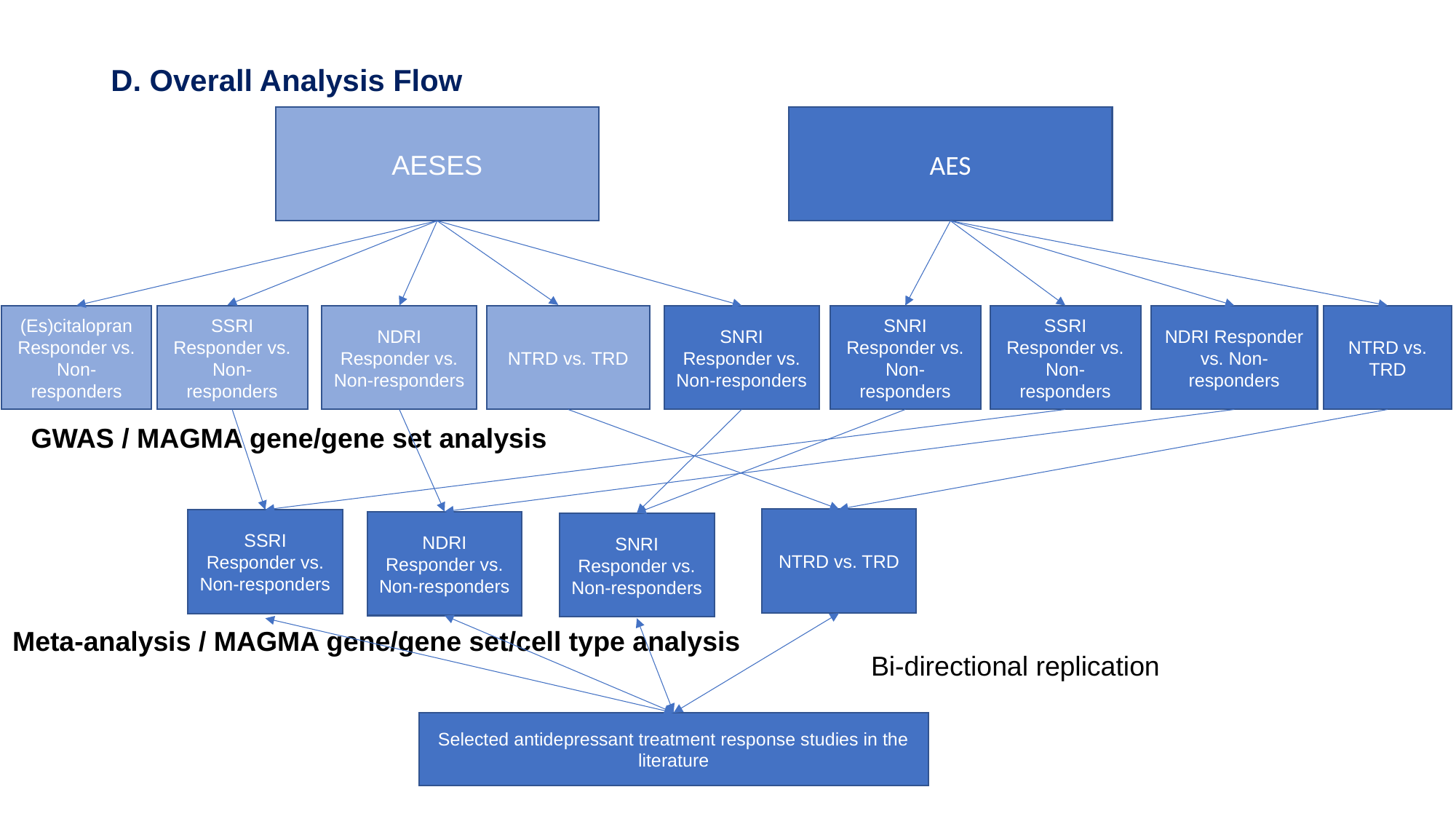

# D. Overall Analysis Flow
AESES
AES
SNRI Responder vs. Non-responders
SNRI
Responder vs. Non-responders
(Es)citalopran Responder vs. Non-responders
NDRI Responder vs. Non-responders
NTRD vs. TRD
NDRI Responder vs. Non-responders
NTRD vs. TRD
SSRI Responder vs. Non-responders
SSRI Responder vs. Non-responders
GWAS / MAGMA gene/gene set analysis
NTRD vs. TRD
SSRI Responder vs. Non-responders
NDRI Responder vs. Non-responders
SNRI Responder vs. Non-responders
Meta-analysis / MAGMA gene/gene set/cell type analysis
Bi-directional replication
Selected antidepressant treatment response studies in the literature
