# Supplementary figures and images for: Genome-wide association studies of antidepressant class response and treatment-resistant depression
Source: Transl Psychiatry. 2020 Oct 26;10:360. doi: 10.1038/s41398-020-01035-6 (PMC7589471; doi:10.1038/s41398-020-01035-6)

S2A

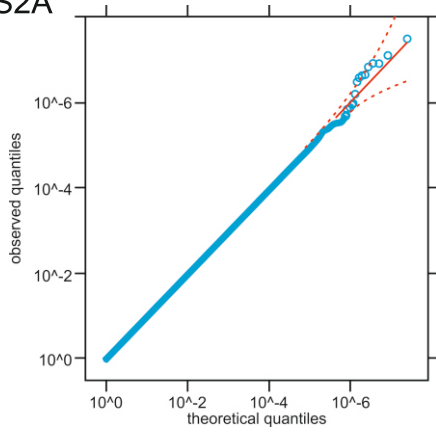

S2B

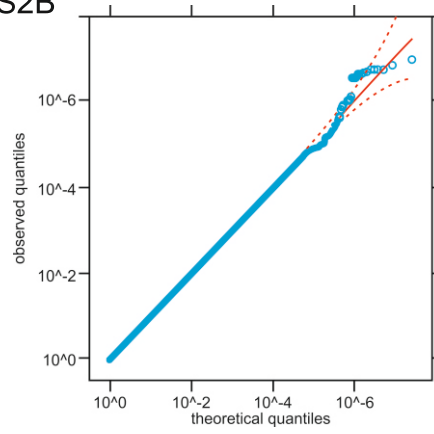

S2C

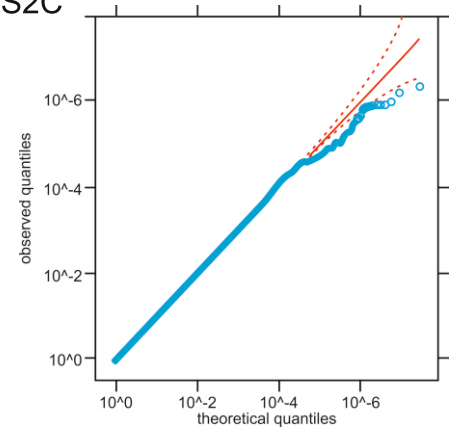

S2D

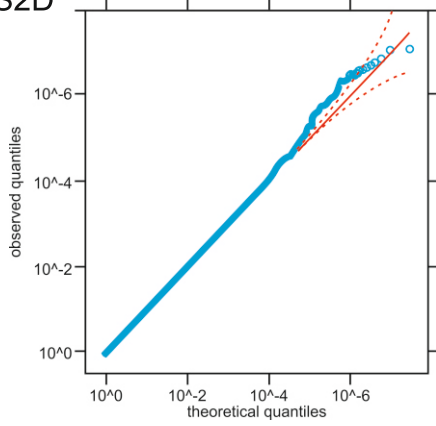

S2E

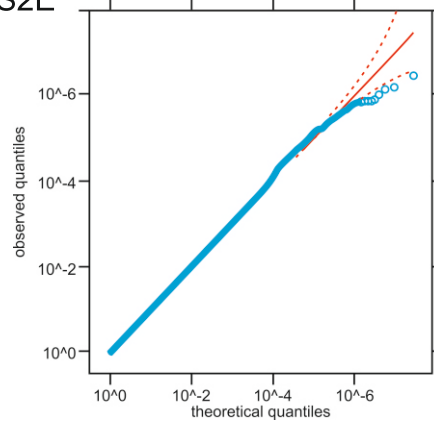

S2F

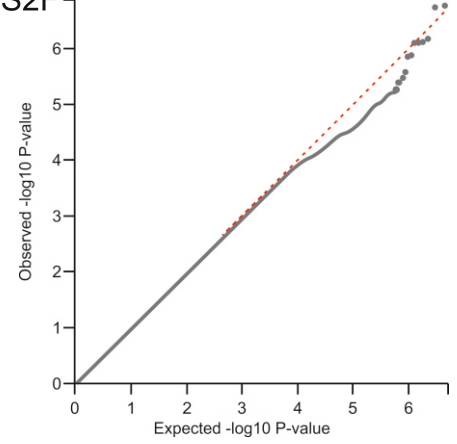

S2G

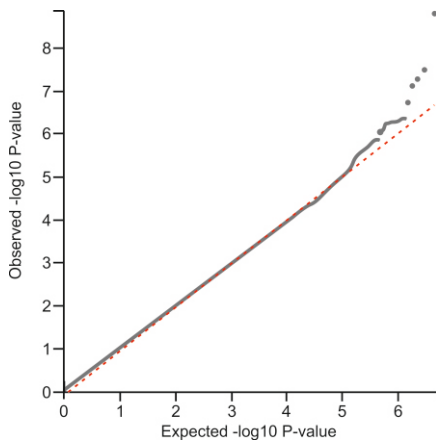

S2H

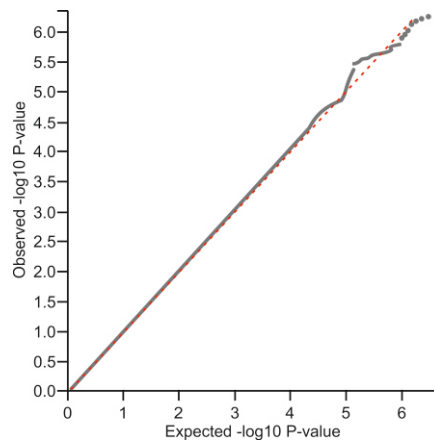

S2I

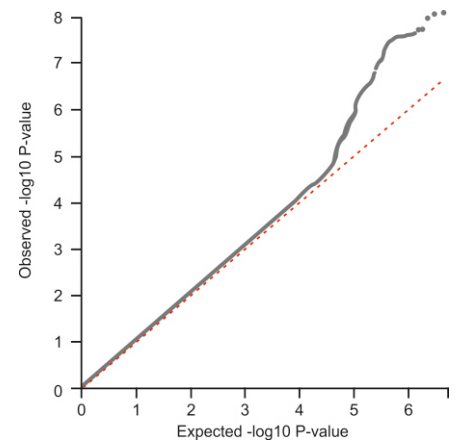

Supplement: Supplementary file 3 — Supplementary Figure S2 [file 41398_2020_1035_MOESM3_ESM.pdf]

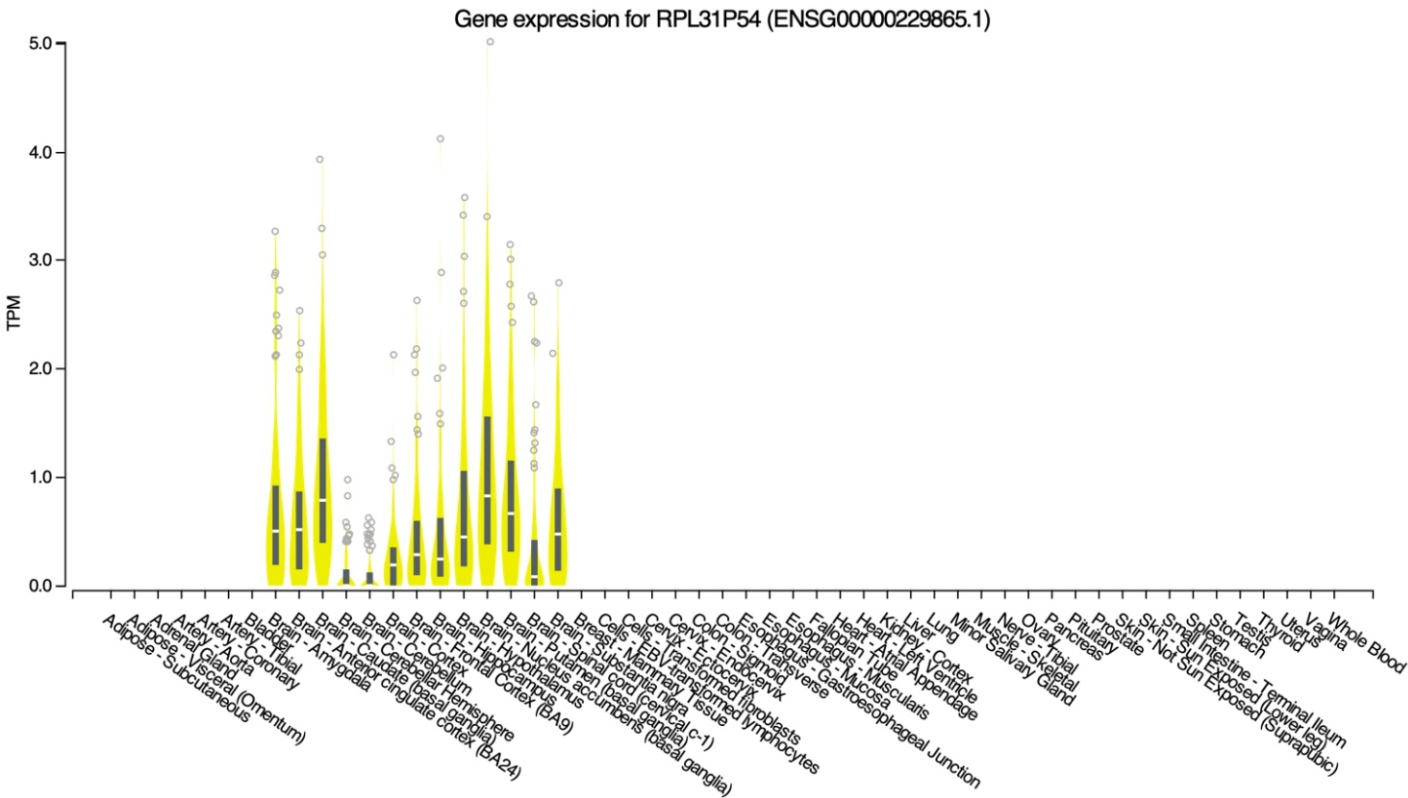

Supplement: Supplementary file 4 — Supplementary Figure S3 [file 41398_2020_1035_MOESM4_ESM.pdf]

S4A

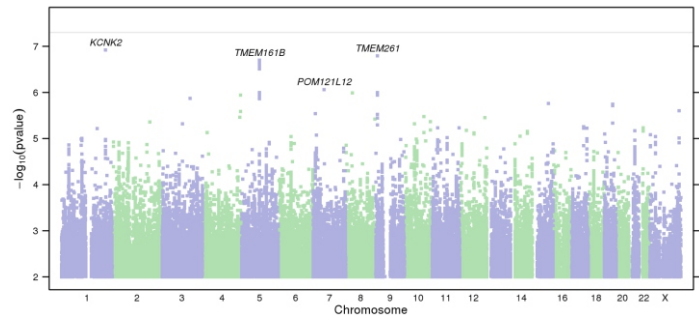

S4B

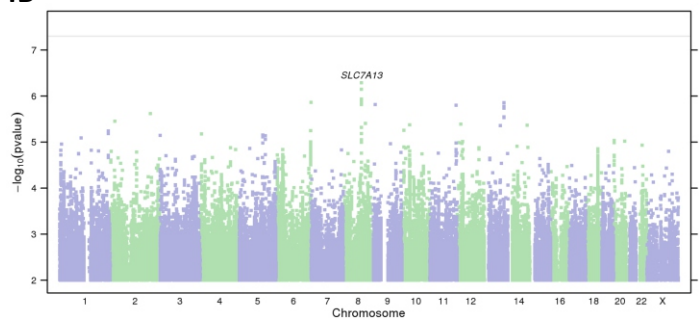

S4C

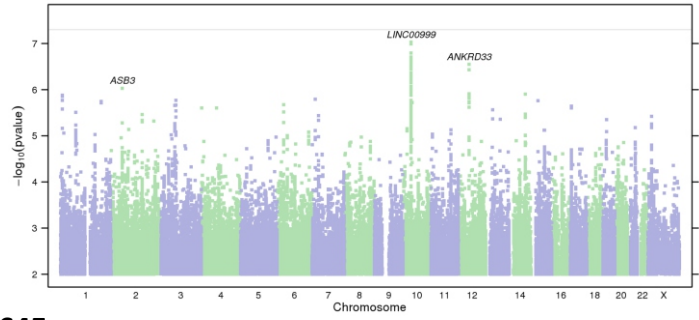

S4D

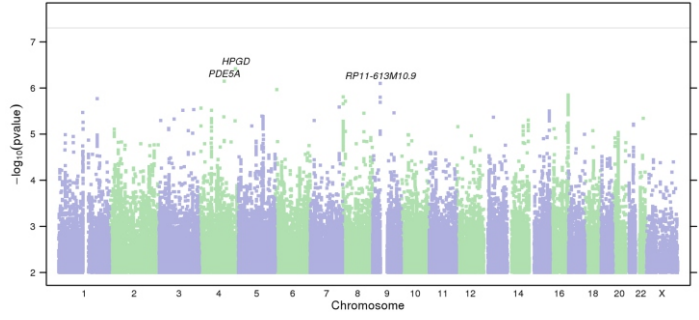

S4E

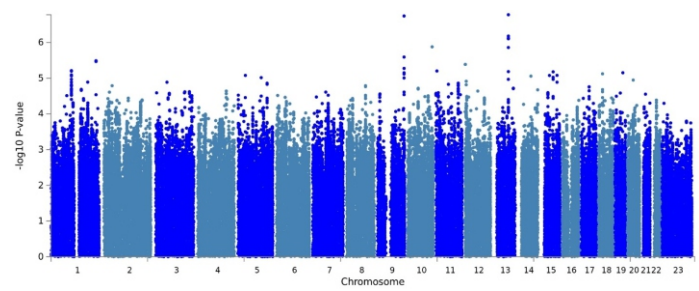

S4F

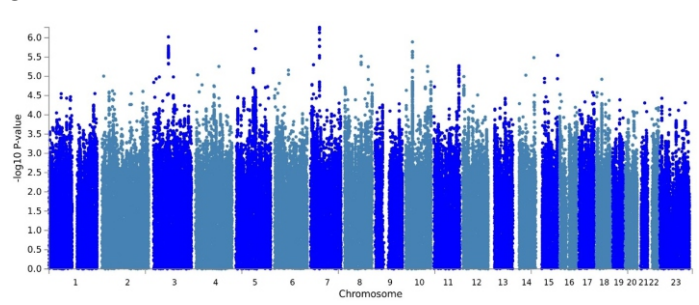

Supplement: Supplementary file 5 — Supplementary Figure S4 [file 41398_2020_1035_MOESM5_ESM.pdf]
