# Supplementary material for: Genome-wide association studies of antidepressant class response and treatment-resistant depression
Source: Transl Psychiatry. 2020 Oct 26;10:360. doi: 10.1038/s41398-020-01035-6 (PMC7589471; doi:10.1038/s41398-020-01035-6)
Supplement: Supplementary file 6 — Supplementary Text [file 41398_2020_1035_MOESM6_ESM.docx]

**Supplementary Text S1. Genome-wide association analysis**

We restricted participants to a set of individuals who had > 97% European ancestry, as determined through an analysis of local ancestry^1^. Briefly, the algorithm first partitions phased genomic data into short windows of about 100 SNPs. Within each window, we used a support vector machine (SVM) to classify individual haplotypes into one of 31 reference populations. The SVM classifications were then fed into a hidden Markov model (HMM) that accounts for switch errors and incorrect assignments and gives probabilities for each reference population in each window. Finally, we used simulated admixed individuals to recalibrate the HMM probabilities so that the reported assignments were consistent with the simulated admixture proportions. The reference population data was derived from public datasets (the Human Genome Diversity Project, HapMap, and 1000 Genomes), as well as 23andMe customers who reported having four grandparents from the same country.

A maximal set of unrelated individuals were chosen for each analysis using a segmental identity-by-descent (IBD) estimation algorithm^47^. Individuals were defined as related if they shared more than 700 cM IBD, including regions where the two individuals share either one or both genomic segments identical-by-descent. This level of relatedness (roughly 20% of the genome) corresponded approximately to the minimal expected sharing between first cousins in an outbred population.

Participant genotype data were imputed against the September 2013 release of 1000 Genomes^48^ Phase1 reference haplotypes, phased with ShapeIt2. We phased and imputed data for each genotyping platform separately. We phased using a phasing tool Finch developed by 23andMe, Inc. which implements the Beagle^49^ haplotype graph-based phasing algorithm, modified to separate the haplotype graph construction and phasing steps. Finch extends the Beagle model to accommodate genotyping error and recombination, to handle cases where there are no consistent paths through the haplotype graph for the individual being phased. We constructed haplotype graphs for European and non-European samples on each 23andMe genotyping platform from a representative sample of genotyped individuals, and then performed out-of-sample phasing of all genotyped individuals against the appropriate graph.

In preparation for imputation, we split phased chromosomes into segments of no more than 10,000 genotyped SNPs, with overlaps of 200 SNPs. Because of large sample size, very small deviations from Hardy–Weinberg equilibrium can give very significant test results. We therefore selected a threshold that tended to reject SNPs with clearly poor intensity plots (i.e. no distinct clusters), but not to reject SNPs that had good clustering or possibly differential missingness (i.e. part of a cluster was being under called), since imputation is tolerant of missing genotypes.

We excluded SNPs with Hardy-Weinberg equilibrium *p* < 10^−20^, call rate < 95%, or with large allele frequency discrepancies compared to European 1000 Genomes reference data. Frequency discrepancies were identified by computing a 2x2 table of allele counts for European 1000 Genomes samples and 2000 randomly sampled 23andMe members with European ancestry and identifying SNPs with a chi squared *p* <10^−15^. We imputed each phased segment against all-ethnicity 1000 Genomes haplotypes (excluding monomorphic and singleton sites) using Minimac2^50^, using 5 rounds and 200 states for parameter estimation.

**Association Test Results**

For quality control of genotyped GWAS results, we excluded SNPs that were only genotyped on our “V1” and/or “V2” platforms due to small sample size, and SNPs on chrM or chrY because many of these are not currently called reliably. Using trio data, we excluded SNPs that failed a test for parent-offspring transmission; specifically, we regressed the child’s allele count against the mean parental allele count and excluded SNPs with fitted *β* < 0.6 and *p* < 10^−20^ for a test of *β* < 1. We excluded SNPs with a Hardy-Weinberg *p* < 10^−20^ in Europeans; or a call rate of < 90%. We also tested genotyped SNPs for genotype date effects and excluded SNPs with *p* < 10^−50^ by ANOVA of SNP genotypes against a factor dividing genotyping date into 20 roughly equal-sized buckets.

For imputed GWAS results, we excluded SNPs with avg.rsq < 0.5 or min.rsq < 0.3 in any imputation batch, as well as SNPs that had strong evidence of an imputation batch effect. The batch effect test is an F test from an ANOVA of the SNP dosage against a factor representing imputation batch; we excluded results with *p* < 10^−50^.

When choosing between imputed and genotyped GWAS results, if either the imputed test passes quality control, or a genotyped test is unavailable, we reported the imputed result; otherwise, we reported the genotyped result.

Across all results, we excluded logistic regression results that did not converge due to complete separation, identified by abs(effect) > 10 or stderr > 10 on the log odds scale.

**Supplementary Text S2. AESES survey and phenotype definition**

The AESES was designed by 23andMe in collaboration with Dr. Steven Hamilton (Institute of Human Genetics, University of California, San Francisco). The questionnaire asked respondents about their use of antidepressants and antipsychotics in the last 5 years (e.g. ‘How well did Wellbutrin^®^ / bupropion work for you?’). The list of drugs included SSRIs (citalopram, escitalopram, fluoxetine, paroxetine, and sertraline), SNRIs (duloxetine, venlafaxine, and desvenlafaxine), NDRI (bupropion), serotonin antagonist and reuptake inhibitor trazodone, and atypical antipsychotics (quetiapine, olanzapine, and aripiprazole). The antidepressant efficacy question in the survey had five possible answers ranging from a great deal (coded as 4 for later reference), a fair amount (3), somewhat (2), a little (1) to not at all (0). The ‘Your Profile and Health History’ survey questioned the participants on their medical history. All participants included in the SNRI responder vs. non-responder analyses self-reported taking antidepressants for depression indication and were of European ancestry. SNRI non-responders were defined as participants who reported efficacy ≤ 1 to at least one SNRI and never reported efficacy ≥ 3 to any SNRI antidepressant, while SNRI-responders were defined as participants who reported efficacy ≥ 3 to at least one SNRI and never reported efficacy ≤ 1 to any SNRI antidepressant.

**Supplementary Text S3. Description of top hits from genome-wide association analysis**

**SSRI GWAS – AES cohort**

The second most significant genomic region (index SNP rs897148, p = 2.1×10−7, OR = 1.168) associated with SSRI response between tribbles pseudokinase 1 (TRIB1) and family with sequence similarity 84 member B (FAM84B) was previously implicated in language performance in older adults^51^ (adjusted for episodic memory) (p = 3.0×10−6). The other suggestive association signal (rs7568567, *p* = 2.5×10^−7^) between Rho family GTPase 3 (*RND3*) and RNA binding motif protein 43 (*RBM43*) may implicate N-myc and STAT interactor (*NMI*) as rs7568567 is in linkage disequilibrium (LD) with rs12328269 (r^2 = 0.672). rs12328269 is an eQTL SNP that correlates with the gene expression of *NMI* (*p*_eQTL_ = 3.1×10^−5^) in lymphoblastoid^52^.

**SNRI GWAS – AES cohort**

Association signals in the SNRI GWAS (SNRI responders vs. non-responders) (index SNP rs193707, *p* = 2.0×10^−7^, OR=0.750) in transmembrane protein 161B (*TMEM161B*) was in LD with rs6870983 (r^2= 0.97) that was previously implicated in body mass index (*p* = 8.0x10^−7^ as described in Locke et al.^53^, and *p* = 4.0x10^-8^ as described in Graff et al.^54^), and rs6452790 (r^2=0.81) that was previously implicated in cognitive function (*p* = 1.0×10^−6^)^55^. rs193707 is also in LD with rs7737179 (r^2 = 0.72) which is correlated with the gene expression level of myocyte enhancer factor 2C (*MEF2C*) (*p*_eQTL_ = 9.6×10^−10^) in whole blood^56^. Another suggestive association signal in 9p24.1 (rs72699890, *p* = 4.2x10^-6^) is also in LD with rs60588302 (r^2 = 0.83) that was implicated in obsessive-compulsive symptoms^57^.

**NDRI GWAS – AES cohort**

In the NDRI GWAS (NDRI responders vs. non-responders) the suggestive association signal (index SNP rs2846674, *p* = 1.39x10^-6^, OR = 1.394) in potassium voltage-gated channel subfamily J member 5 (*KCNJ5*) was in LD with rs66518071 (r^2= 0.518) that was previously implicated in diastolic blood pressure (*p* = 2.2×10^−6^)^58^.

**TRD GWAS – AES cohort**

In the TRD GWAS (NTRD vs. TRD) the suggestive association signal (index SNP rs150245813, *p* = 9.3 × 10^−8^, OR = 0.793) between zinc finger protein 37A (*ZNF37A*) and long intergenic non-protein coding RNA 999 (*LINC00999*) was in LD with a Ser563Arg missense variant in *ZNF37A* (rs146815512, r^2 = 0.960). Furthermore, rs150245813 is also in LD with two eQTL variants (chr10:38465943:I (r^2 = 0.534) that correlates with gene expression level of *RP11-162G10.5* (*p*_eQTL_=4.3×10^−6^) in thyroid^13^ and rs2804669 (r^2 = 0.573) that correlates with gene expression level of *HSD17B7P2* (*p*_eQTL_=1.3 × 10^-33^) in liver^59^. Another suggestive association signal (index SNP rs114431916, *p* = 2.8 × 10^-7^, OR = 24.642, 95% CI [2.913,208.484]) between ankyrin repeat domain 33 (*ANKRD33*) and activin A receptor like type 1 (*ACVRL1*) also implicated an Arg441Lys missense variant in *ANKRD33* (rs149492537, r^2=1.000). The suggestive association signal from 1p36.23 (index SNP rs79066421, *p* = 1.3 × 10^−6^, OR=0.843) between enolase 1 (*ENO1*) and carbonic anhydrase 6 (*CA6*) was also in LD with an eQTL variant rs4609454 (r^2 = 0.509) that correlates with the gene expression level of arginine-glutamic acid dipeptide repeats (*RERE*) (*p*_eQTL_ = 3.2 × 10^−5^) in monocyte22. *RERE* was recently implicated in MDD disease susceptibility^60^, although there may not be the ultimate effector gene as the region may harbor the super-enhancer elements given its many DNA-DNA interactions with many nearby genes. Furthermore, the MDD signal near *RERE* is independent of the signal observed in this study.
