# Supplementary material for: Genome-wide association studies of antidepressant class response and treatment-resistant depression
Source: Transl Psychiatry. 2020 Oct 26;10:360. doi: 10.1038/s41398-020-01035-6 (PMC7589471; doi:10.1038/s41398-020-01035-6)
Supplement: Supplementary file 7 — Supplementary Tables [file 41398_2020_1035_MOESM7_ESM.docx]

**Supplementary Table S1** **Sample size and basic demographic and genomic control inflation factor**

| **Study** | **Group** | **Total** | **Gender** | | **Age** | | | | | **Platform** | | | **Genomic control inflation factor** | | |
| --- | --- | --- | --- | --- | --- | --- | --- | --- | --- | --- | --- | --- | --- | --- | --- |
|  |  |  | **M** | **F** | **(0,30)** | | **(30,45)** | **(45,60)** | **(60,Inf)** | **v1/v2** | **v3** | **v4** | **λ** | **λ_1000_** | **λ_10000_** |
| ***Before removing overlapping participants*** | | | | | | | | | | | | | | | |
| **AES** | ***SSRI responders vs. non-responders*** | | | | | | | | | | | | | | |
|  | Responders | 8491 | 2048 | 6443 | 993 | 2103 | | 2511 | 2884 | 49 | 638 | 7804 |  |  |  |
|  | Non-responders | 4046 | 1117 | 2929 | 728 | 1189 | | 1171 | 958 | 18 | 312 | 3716 | 1.015 | 1.003 | 1.027 |
| **AES** | ***SNRI responders vs. non-responders*** | | | | | | | | | | | | | | |
|  | Responders | 2055 | 451 | 1604 | 111 | 443 | | 747 | 754 | 5 | 176 | 1874 |  |  |  |
|  | Non-responders | 1950 | 504 | 1446 | 195 | 544 | | 664 | 547 | 5 | 172 | 1773 | 1.017 | 1.008 | 1.083 |
| **AES** | ***NDRI responders vs. non-responders*** | | | | | | | | | | | | | | |
|  | Responders | 1616 | 502 | 1114 | 148 | 455 | | 567 | 446 | 10 | 124 | 1482 |  |  |  |
|  | Non-responders | 2068 | 602 | 1466 | 280 | 621 | | 679 | 488 | 9 | 183 | 1876 | 1.01 | 1.006 | 1.058 |
| **AES** | ***NTRD vs. TRD*** | | | | | | | | | | | | | | |
|  | NTRD | 17214 | 4281 | 12933 | 1733 | 4182 | | 5501 | 5798 | 95 | 1323 | 15796 |  |  |  |
|  | TRD | 3168 | 894 | 2274 | 493 | 893 | | 1002 | 780 | 14 | 265 | 2889 | 1.016 | 1.003 | 1.031 |
| **AESES** | ***SNRI responders vs. non-responders*** | | | | | | | | | | | | | | |
|  | Responders | 2547 | 656 | 1891 | 160 | 614 | | 833 | 940 | 37 | 761 | 1749 | 1.013 | 1.007 | 1.07 |
|  | Non-responders | 1567 | 508 | 1059 | 159 | 491 | | 510 | 407 | 18 | 517 | 1032 |  |  |  |
| ***AESES*** | ***NDRI responders vs. non-responders*** | | | | | | |  |  |  |  |  |  |  |  |
|  | *case* | *2675* | *799* | *1876* | *297* | *710* | | *914* | *754* | *46* | *840* | *1789* |  |  |  |
|  | *control* | *1861* | *656* | *1205* | *300* | *569* | | *541* | *451* | *27* | *663* | *1171* | *1.007* | *1.003* | *1.032* |
| ***AESES*** | ***SSRI responders vs. non-responders*** | | | | | | |  |  |  |  |  |  |  |  |
|  | *case* | *6348* | *1770* | *4578* | *671* | *1543* | | *2033* | *2101* | *103* | *1997* | *4248* |  |  |  |
|  | *control* | *3340* | *1229* | *2111* | *666* | *1106* | | *863* | *705* | *44* | *1128* | *2168* | *1.008* | *1.002* | *1.018* |
| ***AESES*** | ***citalopram or escitalopram responders vs. non-responders*** | | | | | | |  |  |  |  |  |  |  |  |
|  | *case* | *2963* | *811* | *2152* | *356* | *806* | | *944* | *857* | *47* | *969* | *1947* |  |  |  |
|  | *control* | *2005* | *690* | *1315* | *438* | *672* | | *516* | *379* | *22* | *661* | *1322* | *1.012* | *1.005* | *1.048* |
| ***AESES*** | ***NTRD vs. TRD*** |  |  |  |  |  | |  |  |  |  |  |  |  |  |
|  | *case* | *7795* | *2204* | *5591* | *799* | *1834* | | *2547* | *2615* | *122* | *2321* | *5352* |  |  |  |
|  | *control* | *1311* | *517* | *794* | *271* | *420* | | *349* | *271* | *14* | *445* | *852* | *1.019* | *1.009* | *1.086* |
| ***After removing overlapping participants*** | | | |  |  |  | |  |  |  |  |  |  |  |  |
| **AES** | ***TRD vs. healthy controls*** | | | | | | | | | | | | | | |
|  | TRD | 2546 | 705 | 1841 | 385 | 736 | | 817 | 608 | 11 | 125 | 2410 | 1.032 | 1.006 | 1.063 |
|  | Healthy controls | 354820 | 184568 | 170252 | 42022 | 75019 | | 102162 | 135617 | 189 | 58 | 354573 |  |  |  |
| **AES** | ***SSRI non-responders vs. healthy controls*** | | | | | | | | | | | | | | |
|  | SSRI Non-responders | 3270 | 897 | 2373 | 573 | 986 | | 974 | 737 | 14 | 139 | 3117 | 1.037 | 1.006 | 1.056 |
|  | Healthy controls | 354704 | 184509 | 170195 | 42007 | 75002 | | 102135 | 135560 | 189 | 58 | 354457 |  |  |  |
| **AES** | ***SNRI non-responders vs. healthy controls*** | | | | | | | | | | | | | | |
|  | SNRI Non-responders | 1561 | 392 | 1169 | 153 | 441 | | 539 | 428 | 4 | 86 | 1471 | 1.015 | 1.005 | 1.047 |
|  | Healthy controls | 354962 | 184634 | 170328 | 42040 | 75044 | | 102200 | 135678 | 189 | 58 | 354715 |  |  |  |
| **AES** | **NDRI non-responders vs. healthy controls** | | | |  |  | |  |  |  |  |  |  |  |  |
|  | NDRI Non-responders | 1651 | 471 | 1180 | 210 | 511 | | 547 | 383 | 8 | 97 | 1546 | 1.012 | 1.004 | 1.037 |
|  | Healthy controls | 354984 | 184644 | 170340 | 42042 | 75048 | | 102209 | 135685 | 189 | 58 | 354737 |  |  |  |
| **AES** | ***NTRD vs. healthy controls*** | | | | | | | | | | | | | | |
|  | NTRD | 13854 | 3441 | 10413 | 1409 | 3456 | | 4467 | 4522 | 52 | 632 | 13170 | 1.079 | 1.003 | 1.03 |
|  | Healthy control | 353118 | 183770 | 169348 | 41801 | 74773 | | 101721 | 134823 | 187 | 57 | 352874 |  |  |  |
| **AES** | ***SNRI responders vs. healthy controls*** | | | | | | | | | | | | | | |
|  | SNRI responders | 1643 | 361 | 1282 | 83 | 359 | | 597 | 604 | 1 | 84 | 1558 | 1.008 | 1.002 | 1.023 |
|  | Healthy controls | 354957 | 184622 | 170335 | 42048 | 75044 | | 102201 | 135664 | 189 | 58 | 354710 |  |  |  |
| **AES** | ***NDRI responders vs. healthy controls*** | | | | | | | | | | | | | | |
|  | NDRI responders | 1296 | 395 | 901 | 123 | 367 | | 461 | 345 | 6 | 61 | 1229 | 0.998 | 0.999 | 0.992 |
|  | Healthy controls | 355007 | 184672 | 170335 | 42056 | 75047 | | 102215 | 135689 | 189 | 58 | 354760 |  |  |  |
| **AES** | ***SSRI responders vs. healthy controls*** | | | | | | | | | | | | | | |
|  | SSRI responders | 6782 | 1638 | 5144 | 798 | 1742 | | 2029 | 2213 | 30 | 294 | 6458 | 1.054 | 1.004 | 1.041 |
|  | Healthy controls | 354161 | 184255 | 169906 | 41929 | 74915 | | 102001 | 135316 | 188 | 58 | 353915 |  |  |  |
| **AES** | ***SSRI responders vs. non-responders*** | | | | | | | | | | | | | | |
|  | Responders | 6782 | 1638 | 5144 | 798 | 1742 | | 2029 | 2213 | 30 | 294 | 6458 | 1.014 | 1.003 | 1.032 |
|  | Non-responders | 3270 | 897 | 2373 | 573 | 986 | | 974 | 737 | 14 | 139 | 3117 |  |  |  |
| **AES** | ***SNRI responders vs. non-responders*** | | | | | | | | | | | | | | |
|  | Responders | 1643 | 361 | 1282 | 83 | 359 | | 597 | 604 | 1 | 84 | 1558 | 1.013 | 1.008 | 1.084 |
|  | Non-responders | 1561 | 392 | 1169 | 153 | 441 | | 539 | 428 | 4 | 86 | 1471 |  |  |  |
| **AES** | ***NDRI responders vs. non-responders*** | | | | | | | | | | | | | | |
|  | Responders | 1296 | 395 | 901 | 123 | 367 | | 461 | 345 | 6 | 61 | 1229 | 1.011 | 1.007 | 1.075 |
|  | Non-responders | 1651 | 471 | 1180 | 210 | 511 | | 547 | 383 | 8 | 97 | 1546 |  |  |  |
| **AES** | ***NTRD vs. TRD*** | | | | | | | | | | | | | | |
|  | NTRD | 13854 | 3441 | 10413 | 1409 | 3456 | | 4467 | 452 | 52 | 632 | 13170 | 1.011 | 1.003 | 1.026 |
|  | TRD | 2546 | 705 | 1841 | 385 | 736 | | 817 | 608 | 11 | 125 | 2410 |  |  |  |
| **AESES** | ***SNRI responders vs. non-responders*** | | | | | | | | | | | | | | |
|  | Responders | 2387 | 617 | 1770 | 218 | 590 | | 841 | 738 | 37 | 761 | 1589 | 1.015 | 1.008 | 1.083 |
|  | Non-responders | 1488 | 492 | 996 | 204 | 483 | | 455 | 346 | 18 | 519 | 951 |  |  |  |
| *AES* Antidepressant Efficacy Survey, *AESES* Antidepressant Efficacy and Side Effects Survey, *NDRI* norepinephrine–dopamine reuptake inhibitor, *NTRD* non-treatment-resistant depression, *SNRI* serotonin-norepinephrine reuptake inhibitor, *SSRI* selective serotonin reuptake inhibitor, *TRD* treatment-resistant depression. Note that analyses reported previously in Li et al., 2016 were *italicized*. | | | | | | | | | | | | | | | |

**Supplementary Table S2: Top hits from GWAS meta-analysis with *p* ≤ 5x10^-4^ together with association statistics from AESES and AES cohort and ANNOVAR annotations (a) NTRD vs. TRD; (b) SSRI responders vs. non-responders; (c) SNRI responders vs. non-responders; and (d) NDRI responders vs. non-responders**

**Supplementary Table S3** **Genetic heritability estimates using LD Score Regression and Genome-wide complex trait analysis**

| **Analysis Group** | **AESES^6^** | |  |  | **AES** | |  |  | **Meta-analysis** | | **Reference** | |
| --- | --- | --- | --- | --- | --- | --- | --- | --- | --- | --- | --- | --- |
|  | **LDSC h^2^ liability (SE)** | ***p*** | **GCTA h^2^ liability (SE)** | ***p*** | **LDSC h^2^ liability (SE)** | ***p*** | **GCTA h^2^ liability (SE)** | ***p*** | **LDSC h^2^ liability (SE)** | ***p*** | **LDSC h^2^ liability (SE)** | ***p*** |
| NDRI responders vs. non-responders | -0.0541 (0.088) | 0.27 | 0.1130 (0.102) | 0.13 | 0.1725  (0.242) | 0.24 | 0.000002 (0.157) | 0.50 | 0.0712  (0.112) | 0.26 |  |  |
| SSRI responders vs. non-responders | 0.0456 (0.050) | 0.18 | 0.0339 (0.050) | 0.25 | -0.0762  (0.070) | 0.14 | 0.070 (0.049) | 0.08 | 0.0389  (0.058) | 0.25 |  |  |
| SNRI responders vs. non-responders | 0.385 (0.195) | 0.02 | 0.000002 (0.120) | 0.50 | 0.0556  (0.223) | 0.40 | 0.187 (0.144) | 0.10 | 0.0147  (0.092) | 0.44 |  |  |
| citalopram or escitalopram responders vs. non-responders | 0.1201 (0.093) | 0.10 | 0.1323 (0.095) | 0.09 | N.D. |  | N.D. |  | N.D. |  |  |  |
| non-TRD vs. TRD | 0.11  (0.100) | 0.13 | 0.000002 (0.076) | 0.50 | 0.2631  (0.368) | 0.24 | 0.0682 (0.041) | 0.05 | 0.0779  (0.04) | 0.03 |  |  |
| NDRI non-responder vs. healthy controls | 0.1492 (0.062) | 0.008 |  |  | 0.2639  (0.068) | 5.20×10^-5^ |  |  |  |  |  |  |
| SSRI non-responders vs. healthy controls | 0.1781 (0.032) | 1.75×10^-8^ |  |  | 0.1318  (0.039) | 3.41×10^-4^ |  |  |  |  |  |  |
| SNRI non-responders vs. healthy controls | N.D. |  |  |  | 0.0483  (0.064) | 0.22 |  |  |  |  |  |  |
| citalopram or escitalopram non-responders vs. healthy controls | 0.1946 (0.064) | 0.001 |  |  | N.D. |  |  |  |  |  |  |  |
| TRD vs. healthy controls | 0.1673 (0.054) | 0.0009 |  |  | 0.1949  (0.044) | 5.42×10^-6^ |  |  |  |  |  |  |
| NDRI responders vs. healthy controls | 0.2211 (0.061) | 0.0001 |  |  | 0.1047  (0.088) | 0.12 |  |  |  |  |  |  |
| SSRI responders vs. healthy controls | 0.1441 (0.020) | 3.77×10^-13^ |  |  | 0.1354  (0.018) | 2.69×10^-14^ |  |  |  |  |  |  |
| SNRI responders vs. healthy controls | N.D. |  |  |  | 0.1226  (0.060) | 0.02 |  |  |  |  |  |  |
| citalopram or escitalopram responders vs. healthy controls | 0.1621 (0.035) | 2.33×10^-6^ |  |  | N.D. |  |  |  |  |  |  |  |
| non-TRD vs. healthy controls | 0.141 (0.015) | 4.92×10^-21^ |  |  | 0.1195  (0.011) | 8.59×10^-28^ |  |  |  |  |  |  |
| PGC MDD |  |  |  |  |  |  |  |  |  |  | 0.1894 (0.035) | 4.39×10^-8^ |
| PGC2 MDD |  |  |  |  |  |  |  |  |  |  | 0.0987 (0.006) | 3.47×10^-59^ |
| PGC BP |  |  |  |  |  |  |  |  |  |  | 0.2848 (0.029) |  |
| PGC SCZ |  |  |  |  |  |  |  |  |  |  | 0.3227 (0.025) |  |
| *AES* Antidepressant Efficacy Survey, *AESES* Antidepressant Efficacy and Side Effects Survey, *BP* bipolar, *GCTA* Genome-wide complex trait analysis, *LDSC* Linkage disequilibrium score regression, *MDD* major depressive disorder, *NDRI* norepinephrine–dopamine reuptake inhibitor, *PGC* Psychiatric Genomics Consortium, *SCZ* schizophrenia, *SNRI* serotonin-norepinephrine reuptake inhibitor, *SSRI* selective serotonin reuptake inhibitor, *TRD* treatment-resistant depression. | | | | | | | | | | | | |

**Supplementary Table S4** **MAGMA gene level association analysis for AES and AESES cohorts (*p* < 0.0001)**

| **Gene** | **CHR** | **START** | **STOP** | **NSNPS** | **N** | ***p*** | **SYMBOL** |
| --- | --- | --- | --- | --- | --- | --- | --- |
| ***AES cohort*** | | | | | | | |
| ***SSRI responders vs. non-responders*** | | | | | | | |
| ENSG00000169252 | 5 | 148,196,156 | 148,218,196 | 91 | 12537 | 3.04E-05 | *ADRB2* |
| ENSG00000074181 | 19 | 15,260,444 | 15,321,792 | 172 | 12537 | 3.09E-05 | *NOTCH3* |
| ENSG00000115084 | 2 | 114,452,588 | 114,524,400 | 234 | 12537 | 4.32E-05 | *SLC35F5* |
| ***SNRI responders vs. non-responders*** | | | | | | | |
| ENSG00000154736 | 21 | 28,280,231 | 28,348,832 | 227 | 4005 | 1.84E-06 | *ADAMTS5* |
| ENSG00000124440 | 19 | 46,790,303 | 46,856,690 | 266 | 4005 | 4.12E-06 | *HIF3A* |
| ENSG00000176971 | 11 | 27,005,628 | 27,028,630 | 100 | 4005 | 4.28E-06 | *FIBIN* |
| ENSG00000112486 | 6 | 167,515,295 | 167,563,184 | 198 | 4005 | 1.08E-05 | *CCR6* |
| ENSG00000140543 | 15 | 89,044,790 | 89,099,906 | 163 | 4005 | 1.96E-05 | *DET1* |
| ENSG00000255633 | 6 | 62,274,008 | 62,294,534 | 154 | 4005 | 2.12E-05 | *MTRNR2L9* |
| ENSG00000107807 | 10 | 102,879,257 | 102,907,545 | 82 | 4005 | 2.28E-05 | *TLX1* |
| ENSG00000156869 | 1 | 100,164,259 | 100,242,187 | 261 | 4005 | 3.12E-05 | *FRRS1* |
| ENSG00000092199 | 14 | 21,667,295 | 21,747,653 | 162 | 4005 | 4.29E-05 | *HNRNPC* |
| ***NDRI responders vs. non-responders*** | | | | | | | |
| ENSG00000087448 | 12 | 27,922,953 | 27,965,973 | 139 | 3684 | 7.35E-06 | *KLHL42* |
| ENSG00000105428 | 19 | 5,445,426 | 5,466,867 | 75 | 3684 | 1.27E-05 | *ZNRF4* |
| ENSG00000061794 | 12 | 27,853,706 | 27,919,228 | 152 | 3684 | 2.54E-05 | *MRPS35* |
| ENSG00000146039 | 6 | 25,744,927 | 25,791,419 | 172 | 3684 | 2.56E-05 | *SLC17A4* |
| ENSG00000164508 | 6 | 25,716,291 | 25,736,790 | 85 | 3684 | 2.58E-05 | *HIST1H2AA* |
| ENSG00000146047 | 6 | 25,717,137 | 25,737,573 | 85 | 3684 | 2.58E-05 | *HIST1H2BA* |
| ENSG00000196569 | 6 | 129,194,342 | 129,847,714 | 1731 | 3684 | 3.31E-05 | *LAMA2* |
| ENSG00000205693 | 12 | 27,905,671 | 27,934,209 | 92 | 3684 | 4.38E-05 | *MANSC4* |
| ENSG00000174236 | 12 | 27,839,428 | 27,860,566 | 39 | 3684 | 4.84E-05 | *REP15* |
| ***NTRD vs. TRD*** | | | | | | | |
| ENSG00000204475 | 6 | 31,546,672 | 31,570,762 | 102 | 20382 | 7.97E-07 | *NCR3* |
| ENSG00000204482 | 6 | 31,543,901 | 31,566,686 | 93 | 20382 | 8.40E-07 | *LST1* |
| ENSG00000227507 | 6 | 31,538,302 | 31,560,299 | 80 | 20382 | 1.89E-06 | *LTB* |
| ENSG00000029725 | 17 | 5,175,558 | 5,299,129 | 437 | 20382 | 3.18E-06 | *RABEP1* |
| ENSG00000108559 | 17 | 5,254,258 | 5,333,480 | 320 | 20382 | 3.44E-06 | *NUP88* |
| ENSG00000162621 | 1 | 74,925,562 | 74,988,298 | 87 | 20382 | 1.94E-05 | *LRRC53* |
| ENSG00000129197 | 17 | 5,312,961 | 5,346,196 | 131 | 20382 | 2.29E-05 | *RPAIN* |
| ENSG00000120913 | 8 | 22,425,792 | 22,465,538 | 101 | 20382 | 4.67E-05 | *PDLIM2* |
| ***AESES cohort*** |  |  |  |  |  |  |  |
| ***SNRI responders vs. non-responders*** | | | | | | | |
| ENSG00000130684 | 20 | 25,654,851 | 25,677,477 | 43 | 4114 | 6.47E-06 | *ZNF337* |
| ENSG00000170191 | 20 | 25,593,571 | 25,604,811 | 30 | 4114 | 1.10E-05 | *NANP* |
| ENSG00000101004 | 20 | 25,433,341 | 25,566,153 | 316 | 4114 | 2.55E-05 | *NINL* |
| ENSG00000089169 | 12 | 113,008,184 | 113,336,686 | 588 | 4114 | 6.19E-05 | *RPH3A* |
| ENSG00000148498 | 10 | 34,398,488 | 35,104,253 | 1622 | 4114 | 8.08E-05 | *PARD3* |
| *AES* Antidepressant Efficacy Survey, *AESES* Antidepressant Efficacy and Side Effects Survey, *CHR* chromosome, *MAGMA* Multi-marker Analysis of GenoMic Annotation, *NDRI* norepinephrine-dopamine reuptake inhibitor, *NSNPS* nonsynonymous SNPs, *NTRD* non-treatment-resistant depression, *SNP* single nucleotide polymorphism, *SNRI* serotonin-norepinephrine reuptake inhibitor, *SSRI* selective serotonin reuptake inhibitor, *TRD* treatment-resistant depression | | | | | | | |

**Supplementary Table S5 MAGMA gene level association using meta-analysis summary association statistics**

| **Gene** | **CHR** | **START** | **STOP** | **NSNPS** | **N** | ***p*** | **SYMBOL** |
| --- | --- | --- | --- | --- | --- | --- | --- |
| ***SSRI responders vs. non-responders*** | | | | | | | |
| ENSG00000101306 | 20 | 30397111 | 30432492 | 85 | 19740 | 3.17E-05 | *MYLK2* |
| ENSG00000088325 | 20 | 30317074 | 30399608 | 138 | 19740 | 3.75E-05 | *TPX2* |
| ENSG00000171552 | 20 | 30242255 | 30321792 | 112 | 19740 | 9.10E-05 | *BCL2L1* |
| ENSG00000162951 | 2 | 80505483 | 80541874 | 74 | 19740 | 9.30E-05 | *LRRTM1* |
| ***SNRI responders vs. non-responders*** | | | | | | | |
| ENSG00000137478 | 11 | 72547790 | 72853306 | 249 | 7079 | 4.19E-05 | *FCHSD2* |
| ENSG00000121060 | 17 | 54965270 | 54991399 | 32 | 7079 | 7.08E-05 | *TRIM25* |
| ***NDRI responders vs. non-responders*** | | | | | | | |
| ENSG00000148513 | 10 | 37404785 | 37683039 | 573 | 7483 | 7.11E-06 | *ANKRD30A* |
| ENSG00000181449 | 3 | 181419714 | 181442221 | 15 | 7483 | 3.32E-05 | *SOX2* |
| ENSG00000107338 | 9 | 37909131 | 38079210 | 422 | 7483 | 4.13E-05 | *SHB* |
| ENSG00000154227 | 15 | 100930600 | 101095200 | 779 | 7483 | 7.23E-05 | *CERS3* |
| ENSG00000156804 | 8 | 124500129 | 124563446 | 178 | 7483 | 8.16E-05 | *FBXO32* |
| ***NTRD vs. TRD*** | | | | | | | |
| ENSG00000186020 | 19 | 37015676 | 37106178 | 198 | 25506 | 2.12E-05 | *ZNF529* |
| ENSG00000179468 | 7 | 142713245 | 142734282 | 25 | 25506 | 4.76E-05 | *OR9A2* |
| ENSG00000106443 | 7 | 11003499 | 11219250 | 549 | 25506 | 6.22E-05 | *PHF14* |
| ENSG00000186765 | 17 | 79485422 | 79514156 | 95 | 25506 | 6.55E-05 | *FSCN2* |
| *CHR* chromosome, *MAGMA* Multi-marker Analysis of GenoMic Annotation, *NDRI* norepinephrine-dopamine reuptake inhibitor, *NSNPS* nonsynonymous single-nucleotide polymorphisms, *NTRD* non-treatment-resistant depression, *SNRI* serotonin-norepinephrine reuptake inhibitor, *SSRI* selective serotonin reuptake inhibitor, *TRD* treatment-resistant depression | | | | | | | |

**Supplementary Table S6 MAGMA gene set enrichment analysis in AES and AESES cohorts (*p* < 0.0005)**

| **N_GENES_** | **BETA** | **BETA_STD** | **SE** | ***p*** | **FULL NAME** |
| --- | --- | --- | --- | --- | --- |
| ***AES cohort*** | | | | | |
| ***SSRI responders vs. non-responders*** | | | | | |
| 88 | 0.41 | 0.03 | 0.09 | 2.54E-06 | Curated gene sets: bhat esr1 targets not via akt1 dn |
| 20 | 0.75 | 0.02 | 0.18 | 1.68E-05 | GO bp: go positive regulation of epidermal cell differentiation |
| 13 | 0.89 | 0.02 | 0.23 | 6.43E-05 | GO bp: go pyrimidine containing compound transmembrane transport |
| 511 | 0.13 | 0.02 | 0.04 | 1.92E-04 | Curated gene sets: martoriati mdm4 targets fetal liver dn |
| 56 | 0.39 | 0.02 | 0.11 | 2.54E-04 | GO bp: go positive regulation of epithelial cell differentiation |
| 32 | 0.49 | 0.02 | 0.15 | 4.91E-04 | GO bp: go positive regulation of epidermis development |
| ***SNRI responders vs. non-responders*** | | | | | |
| 12 | 0.99 | 0.03 | 0.23 | 7.18E-06 | GO mf: go sodium amino acid symporter activity |
| 16 | 0.90 | 0.03 | 0.21 | 7.43E-06 | GO mf: go cation amino acid symporter activity |
| 24 | 0.74 | 0.03 | 0.18 | 3.24E-05 | GO mf: go protein lipid complex binding |
| 12 | 0.81 | 0.02 | 0.23 | 1.72E-04 | Curated gene sets: dorn adenovirus infection 24hr up |
| 122 | 0.24 | 0.02 | 0.07 | 4.23E-04 | GO bp: go amino acid transport |
| ***NDRI responders vs. non-responders*** | | | | | |
| 32 | 0.58 | 0.02 | 0.15 | 5.00E-05 | GO bp: go organ or tissue specific immune response |
| 19 | 0.70 | 0.02 | 0.19 | 1.38E-04 | Curated gene sets: pid epha2 fwd pathway |
| 25 | 0.60 | 0.02 | 0.17 | 2.36E-04 | GO bp: go membrane assembly |
| 22 | 0.71 | 0.02 | 0.21 | 3.00E-04 | GO bp: go innate immune response in mucosa |
| 144 | 0.23 | 0.02 | 0.07 | 3.07E-04 | Curated gene sets: darwiche squamous cell carcinoma up |
| 38 | 1.02 | 0.05 | 0.31 | 4.45E-04 | Curated gene sets: nikolsky breast cancer 1q21 amplicon |
| ***NTRD vs. TRD*** | | | | | |
| 9 | 1.08 | 0.02 | 0.27 | 3.38E-05 | Curated gene sets: reactome activation of chaperone genes by atf6 alpha |
| 48 | 0.47 | 0.02 | 0.12 | 4.04E-05 | Curated gene sets: rashi response to ionizing radiation 3 |
| 42 | 0.42 | 0.02 | 0.12 | 2.87E-04 | Curated gene sets: zhan multiple myeloma cd1 dn |
| 331 | 0.16 | 0.02 | 0.05 | 2.95E-04 | Curated gene sets: rickman metastasis up |
| 7 | 1.10 | 0.02 | 0.33 | 3.83E-04 | Curated gene sets: fardin hypoxia 9 |
| 11 | 0.79 | 0.02 | 0.23 | 4.10E-04 | GO bp: go sequestering of metal ion |
| 13 | 0.70 | 0.02 | 0.21 | 4.15E-04 | GO cc: go chromocenter |
| ***AESES cohort*** |  |  |  |  |  |
| ***SNRI responders vs. non-responders*** | | | | | |
| 548 | 0.14 | 0.02 | 0.03 | 1.38E-05 | Curated gene sets: parent mtor signaling up |
| 437 | 0.15 | 0.02 | 0.04 | 9.75E-05 | GO bp: go inflammatory response |
| 12 | 0.74 | 0.02 | 0.21 | 2.06E-04 | Curated gene sets: croonquist stromal stimulation dn |
| 406 | 0.15 | 0.02 | 0.04 | 2.27E-04 | Curated gene sets: foster tolerant macrophage dn |
| 41 | 0.42 | 0.02 | 0.12 | 2.57E-04 | GO bp: go microtubule polymerization or depolymerization |
| 31 | 0.41 | 0.02 | 0.12 | 3.47E-04 | GO bp: go binding of sperm to zona pellucida |
| 14 | 0.75 | 0.02 | 0.22 | 3.97E-04 | GO bp: go bone maturation |
| *AES* Antidepressant Efficacy Survey, *AESES* Antidepressant Efficacy and Side Effects Survey ,*MAGMA* Multi-marker Analysis of GenoMic Annotation, *NDRI* norepinephrine-dopamine reuptake inhibitor, *NTRD* non-treatment-resistant depression, *SNRI* serotonin-norepinephrine reuptake inhibitor, *SSRI* selective serotonin reuptake inhibitor, *TRD* treatment-resistant depression | | | | | |

**Supplementary Table S7 MAGMA gene set enrichment analysis using meta-analysis summary association statistics (*p* < 0.0005)**

| **NGENES** | **BETA** | **BETA STD** | **SE** | ***p*** | **FULL NAME** |
| --- | --- | --- | --- | --- | --- |
| ***SSRI responders vs. non-responders*** | | | | | |
| 31 | 0.547 | 0.0221 | 0.136 | 2.98E-05 | GO cc: go photoreceptor connecting cilium |
| 66 | 0.401 | 0.0237 | 0.1 | 3.21E-05 | Curated gene sets: kegg glioma |
| 36 | 0.568 | 0.0247 | 0.146 | 5.15E-05 | Curated gene sets: vanharanta uterine fibroid with 7q deletion dn |
| 304 | 0.185 | 0.0232 | 0.0481 | 6.19E-05 | GO bp: go exocytosis |
| 104 | 0.302 | 0.0224 | 0.0803 | 8.42E-05 | Curated gene sets: reactome signaling by ils |
| 28 | 0.526 | 0.0202 | 0.148 | 0.00019054 | GO mf: go fibroblast growth factor receptor binding |
| 73 | 0.332 | 0.0206 | 0.0946 | 0.00022192 | Curated gene sets: hoshida liver cancer survival up |
| 10 | 0.892 | 0.0205 | 0.254 | 0.00022581 | Curated gene sets: biocarta gaba pathway |
| 22 | 0.693 | 0.0236 | 0.198 | 0.00022983 | GO bp: go gamma aminobutyric acid signaling pathway |
| 12 | 0.952 | 0.024 | 0.272 | 0.00023244 | Curated gene sets: reactome gaba a receptor activation |
| 19 | 0.583 | 0.0185 | 0.169 | 0.0002758 | Curated gene sets: sa caspase cascade |
| 220 | 0.196 | 0.021 | 0.0566 | 0.00027741 | GO bp: go regulated exocytosis |
| 53 | 0.388 | 0.0205 | 0.113 | 0.00029579 | Curated gene sets: kegg endometrial cancer |
| 70 | 0.334 | 0.0203 | 0.0999 | 0.00041115 | Curated gene sets: kegg long term depression |
| 79 | 0.312 | 0.0201 | 0.0936 | 0.00043182 | Curated gene sets: reactome response to elevated platelet cytosolic ca2 |
| 72 | 0.312 | 0.0192 | 0.0937 | 0.00044095 | Curated gene sets: kegg melanoma |
| ***SNRI responders vs. non-responders*** | | | | | |
| 12 | 0.998 | 0.0253 | 0.24 | 0.000016286 | Curated gene sets: ding lung cancer mutated frequently |
| 33 | 0.548 | 0.023 | 0.144 | 0.000070728 | GO bp: go peptide hormone processing |
| 25 | 0.595 | 0.0217 | 0.158 | 0.00008293 | Curated gene sets: pid toll endogenous pathway |
| 23 | 0.616 | 0.0216 | 0.165 | 0.000090158 | GO mf: go transferase activity transferring nitrogenous groups |
| 11 | 0.92 | 0.0223 | 0.246 | 0.000090665 | GO bp: go oxaloacetate metabolic process |
| 39 | 0.456 | 0.0208 | 0.126 | 0.00015033 | Curated gene sets: pid erbb4 pathway |
| 6 | 1.23 | 0.022 | 0.343 | 0.00017122 | Curated gene sets: ding lung cancer mutated recurrently |
| 23 | 0.614 | 0.0215 | 0.175 | 0.00022918 | Curated gene sets: reactome growth hormone receptor signaling |
| 25 | 0.595 | 0.0218 | 0.171 | 0.00025375 | Curated gene sets: ding lung cancer mutated significantly |
| 12 | 0.803 | 0.0203 | 0.235 | 0.00031636 | Curated gene sets: croonquist stromal stimulation dn |
| 829 | 0.097 | 0.02 | 0.0288 | 0.00038463 | GO bp: go response to biotic stimulus |
| 14 | 0.75 | 0.0205 | 0.224 | 0.0004094 | GO bp: go jak stat cascade involved in growth hormone signaling pathway |
| ***NDRI responders vs. non-responders*** | | | | | |
| 134 | 0.255 | 0.0214 | 0.0671 | 7.12E-05 | GO bp: go morphogenesis of embryonic epithelium |
| 5 | 1.58 | 0.0258 | 0.423 | 8.85E-05 | Curated gene sets: sharma astrocytoma with nf1 syndrom |
| 129 | 0.241 | 0.0198 | 0.07 | 0.00029567 | GO bp: go tube formation |
| 27 | 0.505 | 0.0191 | 0.148 | 0.00032963 | GO mf: go sulfur compound transmembrane transporter activity |
| 94 | 0.288 | 0.0202 | 0.0869 | 0.00047008 | GO bp: go negative regulation of intracellular protein transport |
| ***NTRD vs. TRD*** | | | | | |
| 134 | 0.246 | 0.0206 | 0.0699 | 0.00021404 | Curated gene sets: provenzani metastasis dn |
| 19 | 0.64 | 0.0203 | 0.184 | 0.00025601 | Curated gene sets: gavin il2 responsive foxp3 targets up |
| 8 | 0.783 | 0.0161 | 0.23 | 0.00033685 | Curated gene sets: sa g2 and m phases |
| 14 | 1.05 | 0.0286 | 0.311 | 0.00035345 | Curated gene sets: nikolsky breast cancer 14q22 amplicon |
| *NDRI* norepinephrine-dopamine reuptake inhibitor, *MAGMA* Multi-marker Analysis of GenoMic Annotation, *NTRD* non-treatment-resistant depression, *SNRI* serotonin-norepinephrine reuptake inhibitor, *SSRI* selective serotonin reuptake inhibitor, *TRD* treatment-resistant depression | | | | | |

**Supplementary Table S8 MAGMA cell type enrichment analysis using meta-analysis summary association statistics**

| **Dataset** | **Cell_type** | **N_GENES_** | **BETA** | **BETA_STD** | **SE** | ***p*** | ***p*_adj.pds_** | ***p*_adj_** |
| --- | --- | --- | --- | --- | --- | --- | --- | --- |
| ***SSRI responders vs. non-responders*** | | | | | | | | |
| Linnarsson_GSE103840_Mouse_Dorsal_horn | Glut_Qrfpr | 15312 | 0.21898 | 0.08444 | 0.065469 | 0.0004 | 0.01 | 0.42 |
| Linnarsson_GSE59739_Mouse_Dorsal_root_ganglion_level2 | PEP2 | 14594 | 0.028652 | 0.062641 | 0.011564 | 0.0066 | 0.05 | 0.94 |
| Linnarsson_GSE59739_Mouse_Dorsal_root_ganglion_level3 | PEP2 | 14594 | 0.025905 | 0.056635 | 0.011254 | 0.0107 | 0.07 | 0.94 |
| Linnarsson_GSE59739_Mouse_Dorsal_root_ganglion_level3 | NF3 | 14594 | 0.019275 | 0.048725 | 0.008603 | 0.0125 | 0.07 | 0.94 |
| GSE89232_Human_Blood | CB_pre_cDC | 14759 | 0.026957 | 0.029358 | 0.026486 | 0.1544 | 0.15 | 0.94 |
| Linnarsson_GSE59739_Mouse_Dorsal_root_ganglion_level2 | NF2.3 | 14594 | 0.019778 | 0.046457 | 0.011628 | 0.0445 | 0.18 | 0.94 |
| Allen_Human_MTG_level1 | Glutamatergic | 18554 | 0.018497 | 0.040725 | 0.011928 | 0.0605 | 0.18 | 0.94 |
| DroNc_Mouse_Hippocampus | exPFC6 | 13010 | 0.48115 | 0.046054 | 0.22075 | 0.0147 | 0.31 | 0.94 |
| Allen_Human_MTG_level2 | Exc_L4.5_FEZF2_SCN4B | 18554 | 0.020451 | 0.048861 | 0.007816 | 0.0044 | 0.33 | 0.94 |
| MouseCellAtlas_Fetal_Brain | Ependymal_cell | 15151 | 0.2316 | 0.024773 | 0.1256 | 0.0326 | 0.35 | 0.94 |
| MouseCellAtlas_Fetal_Brain | Dopaminergic_neurons_ | 15151 | 0.31242 | 0.031171 | 0.18816 | 0.0484 | 0.35 | 0.94 |
| MouseCellAtlas_Fetal_Brain | Microglia | 15151 | 0.16199 | 0.020597 | 0.10361 | 0.0590 | 0.35 | 0.94 |
| GSE98816_Mouse_Brain_Vascular | vEC | 14831 | 0.016688 | 0.028676 | 0.00923 | 0.0353 | 0.36 | 0.94 |
| GSE98816_Mouse_Brain_Vascular | aEC | 14831 | 0.013931 | 0.023809 | 0.009433 | 0.0699 | 0.36 | 0.94 |
| GSE98816_Mouse_Brain_Vascular | EC1 | 14831 | 0.015083 | 0.018346 | 0.012355 | 0.1111 | 0.36 | 0.94 |
| GSE98816_Mouse_Brain_Vascular | EC3 | 14831 | 0.013569 | 0.018012 | 0.011214 | 0.1132 | 0.36 | 0.94 |
| GSE98816_Mouse_Brain_Vascular | EC2 | 14831 | 0.012922 | 0.016744 | 0.011013 | 0.1204 | 0.36 | 0.94 |
| MouseCellAtlas_Fetal_Brain | Macrophage | 15151 | 0.11866 | 0.015476 | 0.092042 | 0.0987 | 0.37 | 0.94 |
| MouseCellAtlas_Fetal_Brain | Radial_glia_Vim_high | 15151 | 0.29614 | 0.028019 | 0.23414 | 0.1030 | 0.37 | 0.94 |
| Linnarsson_GSE59739_Mouse_Dorsal_root_ganglion_level3 | NF2 | 14594 | 0.015693 | 0.03647 | 0.012559 | 0.1057 | 0.39 | 0.94 |
| ***SNRI responders vs. non-responders*** | | | | | | | | |
| Linnarsson_GSE76381_Human_Midbrain | NbML1 | 15512 | 0.20151 | 0.06276 | 0.069075 | 0.0018 | 0.04 | 1.00 |
| Linnarsson_GSE78845_Mouse_Ganglia | Ach1 | 13320 | 0.062488 | 0.06497 | 0.027204 | 0.0108 | 0.06 | 1.00 |
| Linnarsson_GSE76381_Human_Midbrain | RN | 15512 | 0.16706 | 0.047331 | 0.061596 | 0.0033 | 0.08 | 1.00 |
| Allen_Human_MTG_level1 | GABAergic | 18219 | 0.02875 | 0.056275 | 0.015056 | 0.0281 | 0.08 | 1.00 |
| Linnarsson_GSE95752_Mouse_Dentate_gyrus | GABA_Cnr1 | 13015 | 0.10241 | 0.056883 | 0.042008 | 0.0074 | 0.13 | 1.00 |
| Linnarsson_GSE101601_Human_Temporal_cortex | GABA_5 | 18103 | 0.44318 | 0.044817 | 0.21401 | 0.0192 | 0.21 | 1.00 |
| Linnarsson_GSE60361_Mouse_Cortex_Hippocampus_level2 | Int13 | 14591 | 0.076594 | 0.053377 | 0.029466 | 0.0047 | 0.22 | 1.00 |
| Linnarsson_GSE60361_Mouse_Cortex_Hippocampus_level1 | interneurons | 14591 | 0.049544 | 0.035796 | 0.027107 | 0.0338 | 0.24 | 1.00 |
| Linnarsson_GSE59739_Mouse_Dorsal_root_ganglion_level3 | NF2 | 14448 | 0.022933 | 0.053361 | 0.012054 | 0.0286 | 0.31 | 1.00 |
| Linnarsson_GSE59739_Mouse_Dorsal_root_ganglion_level2 | NF2.3 | 14448 | 0.019476 | 0.045799 | 0.011185 | 0.0408 | 0.33 | 1.00 |
| GSE93374_Mouse_Arc_ME_level1 | Neurons2 | 13862 | 0.11199 | 0.033787 | 0.055261 | 0.0214 | 0.43 | 1.00 |
| Linnarsson_GSE101601_Human_Temporal_cortex | GABA_4 | 18103 | 0.24673 | 0.034307 | 0.14407 | 0.0434 | 0.48 | 1.00 |
| Linnarsson_GSE95752_Mouse_Dentate_gyrus | GABA_Lhx6 | 13015 | 0.064021 | 0.03817 | 0.034025 | 0.0300 | 0.51 | 1.00 |
| GSE93374_Mouse_Arc_ME_level1 | ParsTuber2 | 13862 | 0.096002 | 0.028452 | 0.049294 | 0.0257 | 0.51 | 1.00 |
| Linnarsson_GSE103840_Mouse_Dorsal_horn | GABA_Gal_Rspo3 | 15164 | 0.13527 | 0.063336 | 0.064944 | 0.0186 | 0.56 | 1.00 |
| Linnarsson_GSE60361_Mouse_Cortex_Hippocampus_level2 | Int14 | 14591 | 0.055866 | 0.044636 | 0.024744 | 0.0120 | 0.56 | 1.00 |
| Linnarsson_GSE59739_Mouse_Dorsal_root_ganglion_level1 | NF | 14448 | 0.011048 | 0.024932 | 0.011174 | 0.1614 | 0.65 | 1.00 |
| GSE104276_Human_Prefrontal_cortex_all_ages | GABAergic_neurons | 18161 | 0.019299 | 0.030532 | 0.015655 | 0.1088 | 0.65 | 1.00 |
| GSE87544_Mouse_Hypothalamus | GABA8 | 14480 | 0.18835 | 0.048171 | 0.086101 | 0.0144 | 0.66 | 1.00 |
| GSE93374_Mouse_Arc_ME_level2 | Parstuber2A | 13862 | 0.10057 | 0.02972 | 0.048366 | 0.0188 | 0.68 | 1.00 |
| ***NDRI responders vs. non-responders*** | | | | | | | | |
| Allen_Human_MTG_level1 | Glutamatergic | 18550 | 0.029638 | 0.065256 | 0.011987 | 0.0067 | 0.02 | 0.99 |
| Allen_Human_MTG_level2 | Exc_L5.6_FEZF2_ABO | 18550 | 0.029405 | 0.067243 | 0.009013 | 0.0006 | 0.04 | 0.56 |
| Allen_Human_MTG_level2 | Exc_L5.6_RORB_TTC12 | 18550 | 0.028493 | 0.066012 | 0.009347 | 0.0012 | 0.04 | 0.59 |
| Allen_Human_MTG_level2 | Exc_L2.3_LINC00507_FREM3 | 18550 | 0.027047 | 0.062083 | 0.009876 | 0.0031 | 0.08 | 0.99 |
| Allen_Human_MTG_level2 | Exc_L5.6_THEMIS_C1QL3 | 18550 | 0.024511 | 0.057185 | 0.009505 | 0.0050 | 0.09 | 0.99 |
| Allen_Human_MTG_level2 | Exc_L3.5_RORB_FILIP1L | 18550 | 0.024746 | 0.054683 | 0.010061 | 0.0070 | 0.10 | 0.99 |
| GSE67835_Human_Cortex_woFetal | endothelial | 17687 | 0.016484 | 0.024496 | 0.008086 | 0.0208 | 0.15 | 0.99 |
| GSE67835_Human_Cortex | endothelial | 17687 | 0.016751 | 0.024892 | 0.007944 | 0.0175 | 0.16 | 0.99 |
| Allen_Human_MTG_level2 | Exc_L5.6_FEZF2_EFTUD1P1 | 18550 | 0.019319 | 0.044998 | 0.008875 | 0.0148 | 0.18 | 0.99 |
| Allen_Human_MTG_level2 | Exc_L4.5_RORB_FOLH1B | 18550 | 0.019611 | 0.045902 | 0.009546 | 0.0200 | 0.21 | 0.99 |
| Allen_Human_MTG_level2 | Exc_L5.6_THEMIS_DCSTAMP | 18550 | 0.014455 | 0.034173 | 0.007505 | 0.0271 | 0.22 | 0.99 |
| Allen_Human_MTG_level2 | Exc_L5.6_THEMIS_FGF10 | 18550 | 0.014979 | 0.03509 | 0.007954 | 0.0298 | 0.22 | 0.99 |
| Allen_Human_MTG_level2 | Exc_L4.6_FEZF2_IL26 | 18550 | 0.017084 | 0.03771 | 0.009072 | 0.0299 | 0.22 | 0.99 |
| GSE104276_Human_Prefrontal_cortex_all_ages | Microglia | 18481 | 0.014922 | 0.027001 | 0.00837 | 0.0373 | 0.22 | 0.99 |
| GSE87544_Mouse_Hypothalamus | Glu1 | 14622 | 0.14053 | 0.039056 | 0.054652 | 0.0051 | 0.23 | 0.99 |
| Allen_Human_MTG_level2 | Exc_L3.4_RORB_CARM1P1 | 18550 | 0.017203 | 0.039868 | 0.009456 | 0.0344 | 0.23 | 0.99 |
| Linnarsson_GSE101601_Human_Temporal_cortex | Glut_1 | 18246 | 0.41108 | 0.05718 | 0.20812 | 0.0241 | 0.27 | 0.99 |
| Allen_Human_MTG_level2 | Exc_L2_LAMP5_LTK | 18550 | 0.015986 | 0.034061 | 0.009395 | 0.0444 | 0.28 | 0.99 |
| Linnarsson_GSE78845_Mouse_Ganglia | Ach1 | 13429 | 0.049469 | 0.05137 | 0.02846 | 0.0411 | 0.29 | 0.99 |
| Linnarsson_GSE78845_Mouse_Ganglia | Noradrenergic5 | 13429 | 0.054459 | 0.056802 | 0.039252 | 0.0827 | 0.29 | 0.99 |
| *NTRD vs. TRD* | | | | | | | | |
| GSE87544_Mouse_Hypothalamus | Glu11 | 14622 | 0.24907 | 0.077383 | 0.067338 | 0.0001 | 0.01 | 0.13 |
| Linnarsson_GSE78845_Mouse_Ganglia | Ach1 | 13429 | 0.068215 | 0.070836 | 0.028159 | 0.0077 | 0.03 | 0.99 |
| Linnarsson_GSE78845_Mouse_Ganglia | Glutamatergic | 13429 | 0.04661 | 0.045123 | 0.019826 | 0.0094 | 0.03 | 0.99 |
| Allen_Human_LGN_level2 | LGN_Exc_PRKCG_BCHE | 18553 | 0.021005 | 0.04988 | 0.007758 | 0.0034 | 0.04 | 0.99 |
| Allen_Human_LGN_level1 | Glutamatergic | 18553 | 0.018481 | 0.043954 | 0.009802 | 0.0297 | 0.09 | 0.99 |
| MouseCellAtlas_Fetal_Brain | Ependymal_cell | 15149 | 0.30546 | 0.032673 | 0.12796 | 0.0085 | 0.15 | 0.99 |
| Allen_Human_MTG_level2 | Exc_L5.6_FEZF2_ABO | 18553 | 0.023249 | 0.053165 | 0.009075 | 0.0052 | 0.21 | 0.99 |
| Allen_Human_MTG_level2 | Exc_L4.5_FEZF2_SCN4B | 18553 | 0.019498 | 0.046586 | 0.007884 | 0.0067 | 0.21 | 0.99 |
| Allen_Human_MTG_level2 | Exc_L6_FEZF2_OR2T8 | 18553 | 0.017628 | 0.042281 | 0.007424 | 0.0088 | 0.21 | 0.99 |
| Allen_Human_MTG_level2 | Exc_L4.6_FEZF2_IL26 | 18553 | 0.020843 | 0.046006 | 0.009144 | 0.0113 | 0.21 | 0.99 |
| GSE106678_Mouse_Cortex | Ex6 | 15280 | 0.32958 | 0.071425 | 0.12908 | 0.0053 | 0.21 | 0.99 |
| Linnarsson_GSE74672_Mouse_Hypothalamus_level1 | microglia | 15310 | 0.10366 | 0.022974 | 0.058848 | 0.0391 | 0.27 | 0.99 |
| Allen_Human_LGN_level2 | LGN_Exc_BTNL9 | 18553 | 0.012298 | 0.029442 | 0.007839 | 0.0583 | 0.35 | 0.99 |
| Linnarsson_GSE76381_Mouse_Midbrain | NbDA | 15310 | 0.16461 | 0.052258 | 0.078941 | 0.0185 | 0.36 | 0.99 |
| Linnarsson_GSE76381_Mouse_Midbrain | NbM | 15310 | 0.11719 | 0.037591 | 0.061205 | 0.0278 | 0.36 | 0.99 |
| Allen_Mouse_ALM2_level1 | Endothelial | 15526 | 0.008476 | 0.014359 | 0.006406 | 0.0929 | 0.37 | 0.99 |
| Allen_Human_LGN_level2 | LGN_Exc_PRKCG_GRB14 | 18553 | 0.009971 | 0.023484 | 0.007572 | 0.0940 | 0.38 | 0.99 |
| Allen_Human_LGN_level2 | OPC_PDGFRA | 18553 | 0.008362 | 0.013535 | 0.0073 | 0.1260 | 0.38 | 0.99 |
| GSE98816_Mouse_Brain_Vascular | EC2 | 14829 | 0.018037 | 0.02337 | 0.010998 | 0.0505 | 0.38 | 0.99 |
| GSE98816_Mouse_Brain_Vascular | vEC | 14829 | 0.013667 | 0.023484 | 0.00924 | 0.0696 | 0.38 | 0.99 |
| *NDRI* norepinephrine-dopamine reuptake inhibitor, *MAGMA* Multi-marker Analysis of GenoMic Annotation, *NTRD* non-treatment-resistant depression, *SNRI* serotonin-norepinephrine reuptake inhibitor, *SSRI* selective serotonin reuptake inhibitor, *TRD* treatment-resistant depression | | | | | | | | |

**Supplementary Table S9.** Replication results of the reported suggestive associations from GENDEP, MARS, and STAR*D meta-analysis study

|  | **This study** | | | | | | | ***Meta-analysis of the entire GENDEP, MARS, and STAR*D samples*** | | | | | | | | |
| --- | --- | --- | --- | --- | --- | --- | --- | --- | --- | --- | --- | --- | --- | --- | --- | --- |
| **SNP** | **CHR** | **BP** | **A1** | **A2** | ***p*** | **OR** | **Analysis** | **CHR** | **Alleles** | **MAF** | **Beta/OR** | **SE** | ***p*_fixed_** | ***p*_random_** | **Direction** | **Analysis group** |
| rs1517928 | 3 | 65007135 | G | A | 0.001 | 0.91 | TRD | 3 | AG | 0.317 | 0.724 | 0.08 | 3.86E-05 | 3.86E-05 | --- | remission after up to 12 weeks of treatment |
| rs2566785 | 1 | 68604094 | G | A | 0.001 | 1.12 | SNRI | 1 | GA | 0.388 | 0.876424 | 0.03 | 1.76E-05 | 1.76E-05 | --- | percentage improvement over 2 weeks of treatment |
| rs7337666 | 13 | 79073248 | T | G | 0.002 | 0.93 | SSRI | 13 | TG | 0.352 | 1.345 | 0.08 | 8.19E-05 | 8.19E-05 | +++ | remission after up to 12 weeks of treatment |
| rs9523881 | 13 | 93643214 | G | A | 0.002 | 0.88 | SNRI | 13 | GA | 0.173 | 1.438435 | 0.09 | 4.52E-05 | 0.000589 | +++ | remission after up to 12 weeks of treatment |
| rs4716052 | 6 | 16244385 | G | A | 0.004 | 1.11 | NDRI | 6 | AG | 0.351 | 0.8765 | 0.03 | 9.00E-05 | 0.002815 | --- | percentage improvement over 2 weeks of treatment |
| rs4716052 | 6 | 16244385 | G | A | 0.004 | 1.08 | TRD | 6 | AG | 0.351 | 0.8765 | 0.03 | 9.00E-05 | 0.002815 | --- | percentage improvement over 2 weeks of treatment |
| rs3800644 | 7 | 137421386 | T | C | 0.004 | 0.94 | SSRI | 7 | CT | 0.347 | 1.350621 | 0.07 | 2.94E-05 | 0.000575 | +++ | remission after up to 12 weeks of treatment |
| rs388747 | 3 | 120511347 | T | C | 0.006 | 1.11 | NDRI | 3 | CT | 0.323 | 0.877193 | 0.03 | 5.07E-05 | 5.07E-05 | --- | percentage improvement over 12 weeks of treatment |
| rs1673101 | 5 | 87034352 | G | A | 0.006 | 0.91 | NDRI | 5 | AG | 0.333 | 0.8749 | 0.03 | 3.33E-05 | 0.0083 | --- | percentage improvement over 2 weeks of treatment |
| rs7174755 | 15 | 68597127 | T | C | 0.007 | 0.91 | NDRI | 15 | CT | 0.368 | 0.856164 | 0.03 | 9.12E-07 | 0.002348 | --- | percentage improvement over 2 weeks of treatment |
| rs925820 | 3 | 2868988 | T | G | 0.007 | 0.94 | SSRI | 3 | TG | 0.41 | 0.8812 | 0.03 | 9.71E-05 | 9.71E-05 | --- | percentage improvement over 2 weeks of treatment |
| rs3891175 | 6 | 32634467 | T | C | 0.008 | 1.12 | SNRI | 6 | TC | 0.21 | 0.8606 | 0.04 | 5.13E-05 | 5.13E-05 | --- | percentage improvement over 12 weeks of treatment |
| rs2713604 | 3 | 128200459 | T | C | 0.008 | 0.91 | SNRI | 3 | TC | 0.332 | 1.155 | 0.04 | 9.69E-05 | 9.69E-05 | +++ | percentage improvement over 12 weeks of treatment |
| rs3812550 | 9 | 139252879 | G | A | 0.009 | 0.92 | NDRI | 9 | GA | 0.49 | 1.153669 | 0.04 | 5.51E-05 | 0.000569 | +++ | percentage improvement over 2 weeks of treatment |
| rs10174573 | 2 | 193742173 | T | C | 0.009 | 0.90 | SNRI | 2 | CT | 0.425 | 0.639386 | 0.09 | 1.62E-06 | 1.62E-06 | --- | partial response after up to 2 weeks of treatment |
| rs10197851 | 2 | 10485409 | G | A | 0.009 | 0.91 | SNRI | 2 | GA | 0.429 | 1.322401 | 0.07 | 6.61E-05 | 0.0045 | +++ | partial response after up to 2 weeks of treatment |
| rs871696 | 2 | 52856914 | G | A | 0.01 | 0.90 | SNRI | 2 | GA | 0.244 | 0.718391 | 0.08 | 6.72E-05 | 6.72E-05 | --- | remission after up to 12 weeks of treatment |
| rs1909986 | 5 | 4369247 | G | A | 0.01 | 0.91 | NDRI | 5 | GA | 0.225 | 1.37836 | 0.08 | 4.47E-05 | 0.000128 | +++ | partial response after up to 2 weeks of treatment |
| rs6945203 | 7 | 36660730 | G | A | 0.01 | 0.87 | NDRI | 7 | AG | 0.0971 | 1.221 | 0.05 | 9.14E-05 | 9.14E-05 | +++ | percentage improvement over 12 weeks of treatment |
| rs12448844 | 16 | 14263799 | T | C | 0.01 | 0.84 | NDRI | 16 | TC | 0.072 | 2.659 | 0.24 | 5.67E-05 | 0.00237 | + + | remission after up to 12 weeks of treatment |
| rs2648880 | 8 | 129074099 | G | A | 0.02 | 0.94 | SSRI | 8 | GA | 0.237 | 0.870322 | 0.04 | 9.16E-05 | 9.16E-05 | --- | percentage improvement over 2 weeks of treatment |
| rs1348261 | 8 | 106771257 | G | A | 0.02 | 0.95 | SSRI | 8 | AG | 0.479 | 1.303 | 0.07 | 9.03E-05 | 9.03E-05 | +++ | partial response after up to 2 weeks of treatment |
| rs10231884 | 7 | 79084800 | G | A | 0.02 | 1.06 | TRD | 7 | AG | 0.411 | 0.7442 | 0.07 | 2.85E-05 | 0.000444 | --- | remission after up to 12 weeks of treatment |
| rs4530845 | 6 | 55087410 | T | G | 0.02 | 1.10 | SNRI | 6 | TG | 0.232 | 1.375 | 0.08 | 8.25E-05 | 0.000116 | +++ | remission after up to 12 weeks of treatment |
| rs333079 | 1 | 110525630 | G | A | 0.02 | 1.06 | SSRI | 1 | GA | 0.326 | 1.360359 | 0.07 | 3.07E-05 | 3.07E-05 | +++ | remission after up to 12 weeks of treatment |
| rs1909986 | 5 | 4369247 | G | A | 0.02 | 0.93 | TRD | 5 | GA | 0.225 | 1.37836 | 0.08 | 4.47E-05 | 0.000128 | +++ | partial response after up to 2 weeks of treatment |
| rs3812550 | 9 | 139252879 | G | A | 0.02 | 0.92 | SNRI | 9 | GA | 0.49 | 1.153669 | 0.04 | 5.51E-05 | 0.000569 | +++ | percentage improvement over 2 weeks of treatment |
| rs10483225 | 22 | 45331876 | T | C | 0.02 | 1.11 | TRD | 22 | CT | 0.083 | 0.801925 | 0.06 | 9.34E-05 | 0.08179 | --+ | percentage improvement over 2 weeks of treatment |
| rs6601037 | 5 | 178886827 | G | C | 0.02 | 1.09 | SNRI | 5 | CG | 0.431 | 1.147 | 0.04 | 9.38E-05 | 0.001587 | +++ | percentage improvement over 2 weeks of treatment |
| rs17651119 | 5 | 16812638 | T | C | 0.02 | 0.80 | TRD | 5 | TC | 0.0121 | 0.3144 | 0.21 | 1.78E-08 | 0.00314 | --+ | percentage improvement over 12 weeks of treatment |
| rs41170 | 22 | 30421066 | G | A | 0.02 | 0.92 | SNRI | 22 | AG | 0.37 | 1.148 | 0.03 | 1.25E-05 | 0.001621 | +++ | percentage improvement over 2 weeks of treatment |
| rs388747 | 3 | 120511347 | T | C | 0.02 | 1.09 | SNRI | 3 | CT | 0.323 | 0.877193 | 0.03 | 5.07E-05 | 5.07E-05 | --- | percentage improvement over 12 weeks of treatment |
| rs12210761 | 6 | 10176036 | G | A | 0.02 | 1.22 | NDRI | 6 | AG | 0.0401 | 1.936 | 0.16 | 3.17E-05 | 0.0278 | +++ | partial response after up to 2 weeks of treatment |
| rs1586910 | 12 | 91696263 | T | C | 0.02 | 1.07 | TRD | 12 | CT | 0.186 | 0.827815 | 0.04 | 2.56E-05 | 2.56E-05 | -- | percentage improvement over 2 weeks of treatment |
| rs3812550 | 9 | 139252879 | G | A | 0.03 | 0.95 | TRD | 9 | GA | 0.49 | 1.153669 | 0.04 | 5.51E-05 | 0.000569 | +++ | percentage improvement over 2 weeks of treatment |
| rs1431273 | 13 | 109688590 | T | C | 0.03 | 1.09 | SNRI | 13 | CT | 0.344 | 0.883392 | 0.03 | 8.39E-05 | 8.39E-05 | --- | percentage improvement over 2 weeks of treatment |
| rs10509304 | 10 | 70645107 | T | C | 0.03 | 0.80 | SNRI | 10 | TC | 0.0204 | 0.4921 | 0.17 | 3.81E-05 | 0.000281 | --- | percentage improvement over 12 weeks of treatment |
| rs1182102 | 1 | 86830530 | G | A | 0.03 | 0.95 | SSRI | 1 | AG | 0.3 | 1.135 | 0.03 | 9.54E-05 | 0.007119 | +++ | percentage improvement over 2 weeks of treatment |
| rs1517928 | 3 | 65007135 | G | A | 0.03 | 0.92 | SNRI | 3 | AG | 0.317 | 0.724 | 0.08 | 3.86E-05 | 3.86E-05 | --- | remission after up to 12 weeks of treatment |
| rs41170 | 22 | 30421066 | G | A | 0.03 | 1.08 | NDRI | 22 | AG | 0.37 | 1.148 | 0.03 | 1.25E-05 | 0.001621 | +++ | percentage improvement over 2 weeks of treatment |
| rs10484358 | 6 | 16256043 | T | G | 0.03 | 0.91 | TRD | 6 | TG | 0.0884 | 0.7708 | 0.06 | 1.90E-05 | 0.01125 | --- | percentage improvement over 2 weeks of treatment |
| rs10484358 | 6 | 16256043 | T | G | 0.03 | 0.91 | TRD | 6 | TG | 0.0925 | 0.5643 | 0.14 | 3.80E-05 | 0.00211 | --- | partial response after up to 2 weeks of treatment |
| rs17634917 | 19 | 56265664 | G | A | 0.03 | 0.89 | SSRI | 19 | GA | 0.056 | 0.729395 | 0.07 | 3.26E-06 | 1.42E-05 | --- | percentage improvement over 12 weeks of treatment |
| rs17634917 | 19 | 56265664 | G | A | 0.03 | 0.89 | SSRI | 19 | GA | 0.056 | 0.750188 | 0.07 | 3.04E-05 | 3.04E-05 | --- | percentage improvement over 2 weeks of treatment |
| rs4716052 | 6 | 16244385 | G | A | 0.03 | 1.05 | SSRI | 6 | AG | 0.351 | 0.8765 | 0.03 | 9.00E-05 | 0.002815 | --- | percentage improvement over 2 weeks of treatment |
| rs176481 | 7 | 105727925 | G | A | 0.03 | 0.95 | TRD | 7 | AG | 0.377 | 0.7379 | 0.07 | 3.89E-05 | 9.05E-05 | --- | remission after up to 12 weeks of treatment |
| rs10483225 | 22 | 45331876 | T | C | 0.03 | 1.14 | SNRI | 22 | CT | 0.083 | 0.801925 | 0.06 | 9.34E-05 | 0.08179 | --+ | percentage improvement over 2 weeks of treatment |
| rs1431273 | 13 | 109688590 | T | C | 0.03 | 0.93 | NDRI | 13 | CT | 0.344 | 0.883392 | 0.03 | 8.39E-05 | 8.39E-05 | --- | percentage improvement over 2 weeks of treatment |
| rs12055782 | 6 | 128312033 | G | A | 0.03 | 1.06 | TRD | 6 | GA | 0.281 | 1.148106 | 0.03 | 4.53E-05 | 0.000358 | +++ | percentage improvement over 2 weeks of treatment |
| rs1982911 | 19 | 57630475 | G | A | 0.03 | 0.85 | SSRI | 19 | GA | 0.02 | 1.564456 | 0.11 | 3.46E-05 | 0.000592 | +++ | percentage improvement over 2 weeks of treatment |
| rs2156464 | 11 | 68427244 | G | A | 0.04 | 0.91 | SNRI | 11 | AG | 0.187 | 1.231 | 0.05 | 2.00E-05 | 2.00E-05 | ++ | percentage improvement over 2 weeks of treatment |
| rs6682779 | 1 | 216591419 | T | C | 0.04 | 1.32 | NDRI | 1 | CT | 0.015 | 3.821169 | 0.33 | 3.87E-05 | 3.87E-05 | ++ | remission after up to 12 weeks of treatment |
| rs9601248 | 13 | 80169004 | T | C | 0.04 | 1.05 | TRD | 13 | CT | 0.498 | 1.384083 | 0.07 | 2.39E-06 | 2.39E-06 | +++ | remission after up to 12 weeks of treatment |
| rs79880 | 22 | 27931552 | G | A | 0.04 | 0.95 | TRD | 22 | AG | 0.41 | 1.14 | 0.03 | 1.96E-05 | 1.96E-05 | +++ | percentage improvement over 2 weeks of treatment |
| rs6710590 | 2 | 107269417 | G | A | 0.04 | 0.95 | TRD | 2 | AG | 0.314 | 1.329 | 0.07 | 6.72E-05 | 0.00127 | +++ | partial response after up to 2 weeks of treatment |
| rs1100506 | 7 | 124347899 | T | C | 0.04 | 0.96 | SSRI | 7 | CT | 0.473 | 1.311992 | 0.07 | 8.65E-05 | 8.65E-05 | +++ | remission after up to 12 weeks of treatment |
| rs6945203 | 7 | 36660730 | G | A | 0.04 | 1.12 | SNRI | 7 | AG | 0.0971 | 1.221 | 0.05 | 9.14E-05 | 9.14E-05 | +++ | percentage improvement over 12 weeks of treatment |
| rs1924565 | 1 | 58779442 | T | G | 0.04 | 0.93 | NDRI | 1 | TG | 0.31 | 1.155 | 0.04 | 4.79E-05 | 4.79E-05 | +++ | percentage improvement over 12 weeks of treatment |
| rs7731390 | 5 | 131721749 | G | C | 0.04 | 1.14 | SSRI | 5 | GC | 0.046 | 0.725689 | 0.08 | 5.60E-05 | 5.60E-05 | --- | percentage improvement over 12 weeks of treatment |
| rs3800644 | 7 | 137421386 | T | C | 0.05 | 0.95 | TRD | 7 | CT | 0.347 | 1.350621 | 0.07 | 2.94E-05 | 0.000575 | +++ | remission after up to 12 weeks of treatment |
| rs1431273 | 13 | 109688590 | T | C | 0.05 | 0.95 | SSRI | 13 | CT | 0.344 | 0.883392 | 0.03 | 8.39E-05 | 8.39E-05 | --- | percentage improvement over 2 weeks of treatment |
| rs3891175 | 6 | 32634467 | T | C | 0.05 | 1.06 | TRD | 6 | TC | 0.21 | 0.8606 | 0.04 | 5.13E-05 | 5.13E-05 | --- | percentage improvement over 12 weeks of treatment |
| rs1182102 | 1 | 86830530 | G | A | 0.05 | 0.95 | TRD | 1 | AG | 0.3 | 1.135 | 0.03 | 9.54E-05 | 0.007119 | +++ | percentage improvement over 2 weeks of treatment |
| rs17393618 | 3 | 139490193 | G | A | 0.05 | 0.92 | NDRI | 3 | AG | 0.225 | 0.8533 | 0.04 | 1.98E-05 | 1.98E-05 | --- | percentage improvement over 12 weeks of treatment |
| rs6745958 | 2 | 232870178 | G | A | 0.05 | 0.89 | SNRI | 2 | AG | 0.105 | 0.8166 | 0.05 | 2.77E-05 | 2.77E-05 | --- | percentage improvement over 12 weeks of treatment |
| rs1586910 | 12 | 91696263 | T | C | 0.05 | 1.09 | NDRI | 12 | CT | 0.186 | 0.827815 | 0.04 | 2.56E-05 | 2.56E-05 | -- | percentage improvement over 2 weeks of treatment |
| **SNP** | **CHR** | **BP** | **A1** | **A2** | ***p*** | **OR** | **Analysis** | **CHR** | **Alleles** | **MAF** | **Beta/OR** | **SE** | ***p*_fixed_** | ***p*_random_** | Analysis | |
| rs6540437 | 1 | 207823865 | **G** | A | **0.0008** | 1.09 | **TRD** | 1 | **A**G | 0.499974 | 0.69 | 0.0908 | 4.35E-05 | 4.35E-05 | remission after up to 12 weeks of treatment | |
| rs6540437 | 1 | 207823865 | G | A | **0.003** | 1.11 | **NDRI** | 1 | AG | 0.499974 | 0.69 | 0.0908 | 4.35E-05 | 4.35E-05 | remission after up to 12 weeks of treatment | |
| rs5750428 | 22 | 37794269 | T | C | 0.004 | 0.90 | SNRI | 22 | TC | 0.39484 | -0.1751 | 0.043 | 4.67E-05 | 0.0001 | percentage improvement over 12 weeks of treatment | |
| rs370605 | 5 | 100721491 | T | C | 0.004 | 0.89 | NDRI | 5 | CT | 0.219442 | 0.1905 | 0.0484 | 8.43E-05 | 8.43E-05 | Results of meta-analysis of early improvement among SSRI-treated subjects in GENDEP and STAR*D. (a) percentage improvement over 2 weeks of treatment | |
| rs3762999 | 5 | 150469426 | T | C | 0.004 | 0.91 | SNRI | 5 | TC | 0.441714 | -0.1642 | 0.0385 | 1.99E-05 | 1.99E-05 | Results of meta-analysis of early improvement among SSRI-treated subjects in GENDEP and STAR*D. (a) percentage improvement over 2 weeks of treatment | |
| rs2415062 | 15 | 70209553 | G | A | 0.005 | 0.91 | SNRI | 15 | AG | 0.422059 | 0.1513 | 0.0386 | 8.72E-05 | 0.004844 | percentage improvement over 12 weeks of treatment | |
| rs370605 | 5 | 100721491 | T | C | 0.005 | 0.92 | TRD | 5 | CT | 0.219442 | 0.1905 | 0.0484 | 8.43E-05 | 8.43E-05 | Results of meta-analysis of early improvement among SSRI-treated subjects in GENDEP and STAR*D. (a) percentage improvement over 2 weeks of treatment | |
| rs1673101 | 5 | 87034352 | G | A | 0.006 | 0.91 | NDRI | 5 | AG | 0.331215 | -0.1877 | 0.0408 | 4.18E-06 | 4.18E-06 | Results of meta-analysis of early improvement among SSRI-treated subjects in GENDEP and STAR*D. (a) percentage improvement over 2 weeks of treatment | |
| rs8012941 | 14 | 63322347 | G | A | 0.006 | 1.07 | TRD | 14 | GA | 0.497517 | 1.538698 | 0.0939 | 4.48E-06 | 4.48E-06 | remission after up to 12 weeks of treatment | |
| rs7742883 | 6 | 16258216 | G | A | 0.007 | 0.93 | TRD | 6 | GA | 0.392921 | -0.1754 | 0.0396 | 9.59E-06 | 9.59E-06 | Results of meta-analysis of early improvement among SSRI-treated subjects in GENDEP and STAR*D. (a) percentage improvement over 2 weeks of treatment | |
| rs7174755 | 15 | 68597127 | T | C | 0.007 | 0.91 | NDRI | 15 | CT | 0.369048 | -0.2048 | 0.0397 | 2.53E-07 | 2.60E-05 | Results of meta-analysis of early improvement among SSRI-treated subjects in GENDEP and STAR*D. (a) percentage improvement over 2 weeks of treatment | |
| rs28539249 | 9 | 139240630 | T | C | 0.007 | 1.10 | NDRI | 9 | CT | 0.427641 | 0.2211 | 0.0547 | 5.28E-05 | 5.28E-05 | Results of meta-analysis of early improvement among SSRI-treated subjects in GENDEP and STAR*D. (a) percentage improvement over 2 weeks of treatment | |
| rs7742883 | 6 | 16258216 | G | A | 0.007 | 0.91 | NDRI | 6 | GA | 0.392921 | -0.1754 | 0.0396 | 9.59E-06 | 9.59E-06 | Results of meta-analysis of early improvement among SSRI-treated subjects in GENDEP and STAR*D. (a) percentage improvement over 2 weeks of treatment | |
| rs3891175 | 6 | 32634467 | T | C | 0.008 | 1.12 | SNRI | 6 | TC | 0.21819 | -0.1797 | 0.0461 | 9.89E-05 | 9.89E-05 | percentage improvement over 12 weeks of treatment | |
| rs4342663 | 9 | 9422159 | T | C | 0.009 | 1.07 | TRD | 9 | TC | 0.444045 | 1.436782 | 0.0824 | 1.09E-05 | 1.09E-05 | Results of meta-analysis of early partial response among SSRI-treated subjects in GENDEP and STAR*D. (b) partial response after up to 2 weeks of treatment | |
| rs2060609 | 14 | 43338730 | G | A | 0.010 | 1.09 | SSRI | 14 | AG | 0.127787 | 1.7057 | 0.1348 | 7.41E-05 | 0.01876 | Results of meta-analysis of early partial response among SSRI-treated subjects in GENDEP and STAR*D. (b) partial response after up to 2 weeks of treatment | |
| rs9993534 | 4 | 113533796 | T | C | 0.01 | 0.88 | SNRI | 4 | CT | 0.156544 | 1.6021 | 0.121 | 9.84E-05 | 9.84E-05 | Results of meta-analysis of early partial response among SSRI-treated subjects in GENDEP and STAR*D. (b) partial response after up to 2 weeks of treatment | |
| rs7166565 | 15 | 99489935 | G | A | 0.01 | 0.92 | NDRI | 15 | AG | 0.429186 | 0.1566 | 0.038 | 3.67E-05 | 3.67E-05 | percentage improvement over 12 weeks of treatment | |
| rs3846456 | 4 | 96360786 | G | A | 0.01 | 1.07 | SSRI | 4 | GA | 0.276341 | 1.499925 | 0.0986 | 3.93E-05 | 3.93E-05 | Results of meta-analysis of early partial response among SSRI-treated subjects in GENDEP and STAR*D. (b) partial response after up to 2 weeks of treatment | |
| rs904759 | 5 | 1736837 | G | A | 0.01 | 0.92 | NDRI | 5 | AG | 0.306596 | 0.6601 | 0.0956 | 1.39E-05 | 1.39E-05 | remission after up to 12 weeks of treatment | |
| rs12640095 | 4 | 47145034 | G | A | 0.01 | 0.89 | SNRI | 4 | GA | 0.179416 | 0.2191 | 0.051 | 1.71E-05 | 0.01385 | Results of meta-analysis of early improvement among SSRI-treated subjects in GENDEP and STAR*D. (a) percentage improvement over 2 weeks of treatment | |
| rs11685085 | 2 | 119961971 | T | C | 0.01 | 1.07 | TRD | 2 | TC | 0.364753 | 0.702247 | 0.0906 | 9.61E-05 | 9.61E-05 | Results of meta-analysis of early partial response among SSRI-treated subjects in GENDEP and STAR*D. (b) partial response after up to 2 weeks of treatment | |
| rs6540437 | 1 | 207823865 | G | A | **0.01** | 1.05 | **SSRI** | 1 | AG | 0.499974 | 0.69 | 0.0908 | 4.35E-05 | 4.35E-05 | remission after up to 12 weeks of treatment | |
| rs17714187 | 12 | 91469910 | G | C | 0.02 | 0.88 | NDRI | 12 | GC | 0.119097 | -0.2673 | 0.0608 | 1.11E-05 | 2.02E-05 | Results of meta-analysis of early improvement among SSRI-treated subjects in GENDEP and STAR*D. (a) percentage improvement over 2 weeks of treatment | |
| rs17061180 | 3 | 59756690 | T | C | 0.02 | 0.94 | TRD | 3 | CT | 0.417572 | 1.4324 | 0.0877 | 4.22E-05 | 4.22E-05 | Results of meta-analysis of early partial response among SSRI-treated subjects in GENDEP and STAR*D. (b) partial response after up to 2 weeks of treatment | |
| rs10231884 | 7 | 79084800 | G | A | 0.02 | 1.06 | TRD | 7 | AG | 0.374556 | 0.6981 | 0.0908 | 7.53E-05 | 7.53E-05 | remission after up to 12 weeks of treatment | |
| rs311786 | 19 | 11568522 | G | A | 0.02 | 0.92 | TRD | 19 | GA | 0.185867 | 0.2444 | 0.0568 | 1.66E-05 | 1.66E-05 | percentage improvement over 12 weeks of treatment | |
| rs17692896 | 19 | 33089431 | G | A | 0.02 | 0.85 | SNRI | 19 | AG | 0.073918 | -0.4395 | 0.0887 | 7.15E-07 | 0.009604 | Results of meta-analysis of early improvement among SSRI-treated subjects in GENDEP and STAR*D. (a) percentage improvement over 2 weeks of treatment | |
| rs17692896 | 19 | 33089431 | G | A | 0.02 | 0.85 | SNRI | 19 | AG | 0.061934 | 0.4407 | 0.2059 | 6.89E-05 | 6.89E-05 | Results of meta-analysis of early partial response among SSRI-treated subjects in GENDEP and STAR*D. (b) partial response after up to 2 weeks of treatment | |
| rs2073370 | 21 | 35260481 | T | C | 0.02 | 1.05 | SSRI | 21 | TC | 0.435603 | -0.1757 | 0.0401 | 1.17E-05 | 1.17E-05 | percentage improvement over 12 weeks of treatment | |
| rs10416595 | 19 | 9102065 | G | A | 0.02 | 0.95 | SSRI | 19 | AG | 0.330006 | 1.4585 | 0.0966 | 9.28E-05 | 9.28E-05 | remission after up to 12 weeks of treatment | |
| rs7742883 | 6 | 16258216 | G | A | 0.02 | 0.95 | SSRI | 6 | GA | 0.392921 | -0.1754 | 0.0396 | 9.59E-06 | 9.59E-06 | Results of meta-analysis of early improvement among SSRI-treated subjects in GENDEP and STAR*D. (a) percentage improvement over 2 weeks of treatment | |
| rs17691515 | 16 | 20861072 | C | A | 0.02 | 1.08 | SSRI | 16 | AC | 0.128327 | 1.7046 | 0.1355 | 8.27E-05 | 8.27E-05 | Results of meta-analysis of early partial response among SSRI-treated subjects in GENDEP and STAR*D. (b) partial response after up to 2 weeks of treatment | |
| rs3112954 | 2 | 180496360 | T | C | 0.02 | 1.08 | SSRI | 2 | CT | 0.101527 | 0.254 | 0.0643 | 7.92E-05 | 7.92E-05 | Results of meta-analysis of early improvement among SSRI-treated subjects in GENDEP and STAR*D. (a) percentage improvement over 2 weeks of treatment | |
| rs12210761 | 6 | 10176036 | G | A | 0.02 | 1.22 | NDRI | 6 | AG | 0.067661 | 2.4458 | 0.1981 | 6.32E-06 | 0.007691 | Results of meta-analysis of early partial response among SSRI-treated subjects in GENDEP and STAR*D. (b) partial response after up to 2 weeks of treatment | |
| rs9533786 | 13 | 44763117 | C | A | 0.02 | 0.92 | SNRI | 13 | CA | 0.398414 | 0.1584 | 0.0398 | 6.81E-05 | 6.81E-05 | Results of meta-analysis of early improvement among SSRI-treated subjects in GENDEP and STAR*D. (a) percentage improvement over 2 weeks of treatment | |
| rs9993534 | 4 | 113533796 | T | C | 0.03 | 0.92 | TRD | 4 | CT | 0.156544 | 1.6021 | 0.121 | 9.84E-05 | 9.84E-05 | Results of meta-analysis of early partial response among SSRI-treated subjects in GENDEP and STAR*D. (b) partial response after up to 2 weeks of treatment | |
| rs379600 | 3 | 148444485 | G | A | 0.03 | 0.84 | TRD | 3 | AG | 0.038052 | -0.9038 | 0.2219 | 4.64E-05 | 0.000277 | Results of meta-analysis of early improvement among SSRI-treated subjects in GENDEP and STAR*D. (a) percentage improvement over 2 weeks of treatment | |
| rs10509304 | 10 | 70645107 | T | C | 0.03 | 0.80 | SNRI | 10 | TC | 0.020325 | -0.907 | 0.2123 | 1.93E-05 | 0.01722 | percentage improvement over 12 weeks of treatment | |
| rs7597171 | 2 | 157470262 | T | C | 0.03 | 1.07 | TRD | 2 | CT | 0.206037 | 0.6376 | 0.1109 | 4.95E-05 | 0.001382 | remission after up to 12 weeks of treatment | |
| rs10484358 | 6 | 16256043 | T | G | 0.03 | 0.91 | TRD | 6 | TG | 0.082711 | -0.4258 | 0.0884 | 1.46E-06 | 1.46E-06 | Results of meta-analysis of early improvement among SSRI-treated subjects in GENDEP and STAR*D. (a) percentage improvement over 2 weeks of treatment | |
| rs17634917 | 19 | 56265664 | G | A | 0.03 | 0.89 | SSRI | 19 | GA | 0.057395 | -0.3761 | 0.0829 | 5.75E-06 | 5.75E-06 | percentage improvement over 12 weeks of treatment | |
| rs2377360 | 1 | 217200883 | G | A | 0.04 | 1.09 | SNRI | 1 | GA | 0.26324 | 1.6 | 0.1057 | 8.77E-06 | 0.01032 | remission after up to 12 weeks of treatment | |
| rs1525293 | 7 | 70473005 | T | C | 0.04 | 0.93 | NDRI | 7 | TC | 0.340333 | 0.58682 | 0.1126 | 2.19E-06 | 2.19E-06 | remission after up to 12 weeks of treatment | |
| rs1500292 | 16 | 59745634 | T | C | 0.05 | 0.92 | NDRI | 16 | CT | 0.28351 | -0.1688 | 0.0412 | 4.20E-05 | 0.06468 | percentage improvement over 12 weeks of treatment | |
| rs7258661 | 19 | 56691601 | C | A | 0.05 | 0.94 | SSRI | 19 | AC | 0.130493 | 0.3412 | 0.0825 | 3.53E-05 | 0.0469 | Results of meta-analysis of early improvement among SSRI-treated subjects in GENDEP and STAR*D. (a) percentage improvement over 2 weeks of treatment | |
| rs9859077 | 3 | 101136402 | G | C | 0.05 | 0.93 | NDRI | 3 | CG | 0.341732 | 0.1618 | 0.0393 | 3.74E-05 | 0.00043 | percentage improvement over 12 weeks of treatment | |
| rs17133444 | 7 | 138742775 | T | C | 0.05 | 0.91 | NDRI | 7 | TC | 0.168223 | -0.2676 | 0.0682 | 8.60E-05 | 8.60E-05 | percentage improvement over 12 weeks of treatment | |
| rs379600 | 3 | 148444485 | G | A | 0.05 | 0.87 | SSRI | 3 | AG | 0.038052 | -0.9038 | 0.2219 | 4.64E-05 | 0.000277 | Results of meta-analysis of early improvement among SSRI-treated subjects in GENDEP and STAR*D. (a) percentage improvement over 2 weeks of treatment | |
| rs3891175 | 6 | 32634467 | T | C | 0.05 | 1.06 | TRD | 6 | TC | 0.21819 | -0.1797 | 0.0461 | 9.89E-05 | 9.89E-05 | percentage improvement over 12 weeks of treatment | |
| rs16855691 | 1 | 232085485 | C | A | 0.05 | 0.79 | SNRI | 1 | AC | 0.029673 | -0.6681 | 0.168 | 6.96E-05 | 6.96E-05 | percentage improvement over 12 weeks of treatment | |
| TRD: NTRD vs. TRD; SSRI: SSRI responders vs. non-responders; NDRI: NDRI responders vs. non-responders; SNRI: SNRI responders vs. non-responders | | | | | | | | | | | | | | | | |

**Supplementary Table S10. Replication results of the reported suggestive association from Fabbri et al (GENDEP and STAR*D meta-analysis) study**
